# Tartrate‐Assisted Ionic Layer Epitaxy for General Synthesis of 2D Nanostructures

**DOI:** 10.1002/smtd.202501507

**Published:** 2025-11-02

**Authors:** Ziyi Zhang, Derui Wang, Maciej P. Polak, Corey Carlos, Yutao Dong, Dane Morgan, Xudong Wang

**Affiliations:** ^1^ Department of Materials Science and Engineering University of Wisconsin‐Madison Madison Wisconsin 53706 USA

**Keywords:** 2D materials, high‐throughput synthesis, ionic layer epitaxy, large language model

## Abstract

The synthesis of ultrathin 2D nanomaterials with regular shapes and uniform thickness remains a challenge despite their intriguing properties. In this paper, a facile and generic strategy for the growth of versatile ultrathin nanosheets using tartrate‐assisted ionic layer epitaxy (ILE) at the air‐water interface is presented. Metal ions, stabilized by tartrate coordination, nucleate within the electrical double layer beneath a self‐assembled monolayer. This method facilitates the growth of micro‐sized hexagonal to wafer‐scale continuous nanosheets from 26 metal elements, including single and multi‐element compositions, with high precursor utilization via solution recycling. Furthermore, recognizing the need for efficient quality assessment, the successful application of a multimodal Large Language Model (LLM) for rapid and consistent evaluation of nanosheet quality from microscopy images, achieving performance comparable to human experts is demonstrated. This combined approach demonstrates a sustainable and high‐throughput path for both 2D nanomaterial synthesis and automated quality assessment, accelerating materials discovery.

## Graphical abstract

A general and sustainable synthesis of ultrathin 2D nanosheets from a vast library of 26 single‐ and multi‐element compositions is achieved using tartrate‐assisted ionic layer epitaxy. This high‐throughput method can be coupled with a multimodal large language model for automated, expert‐level quality assessment, creating a new pathway to the discovery of advanced 2D materials.

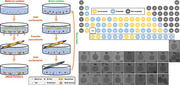

## 1 Introduction

Ultrathin 2D nanosheets made of materials with layered crystalline structures, such as graphene,^[^ ^1^ ^]^ transition metal dichalcogenides,^[^ ^2^ ^]^ and layered double hydroxides^[^ ^3^ ^]^ have attracted extensive attention due to their intriguing chemical, physical and mechanical properties associated with their atomic thickness.^[^ ^4^, ^5^, ^6^, ^7^ ^]^ The preparation of these 2D nanomaterials has often been limited to exploiting naturally layered materials, known as van der Waals solids.^[^ ^4^ ^]^ Typically, naturally layered nanosheets (NSs) have been prepared by the top‐down multistep exfoliation process,^[^ ^2^, ^5^ ^]^ which often yields NSs with a wide distribution of thickness and size. It remains a practical challenge to achieve a convenient synthesis of ultrathin crystalline NSs with uniform thickness and regular shape. Furthermore, the lack of robust mechanisms for the bottom‐up synthesis of 2D nanomaterials from non‐layered materials impedes further exploring the nontrivial properties and advanced applications of 2D nanomaterials.

Inspired by the template regulation effect of protein assemblies in biomineralization,^[^ ^6^, ^7^ ^]^ a few synthesis techniques have been developed adopting a soft template of self‐assembled amphiphilic molecules to control the anisotropic growth of 2D nanomaterials.^[^ ^8^, ^9^, ^10^ ^]^ The effective suppression of bulk nucleation is a key advantage of this method, enabling the synthesis of high‐purity, uniform 2D nanosheets and allowing for a sustainable, high‐throughput process via solution recycling. Among these techniques, ionic layer epitaxy (ILE) is a typical example that utilizes the ionized head groups in an amphiphilic monolayer at the air‐water interface to generate an electrical double layer (EDL) as a confined regime for the nucleation and growth of ultrathin 2D nanocrystals.^[^ ^10^, ^11^, ^12^ ^]^ ILE has been successful in synthesizing a few nanometers‐thick, tens of micron‐sized (size here refers to lateral in‐plane dimensions) single‐crystalline ZnO NSs.^[^ ^11^ ^]^ Studies exploring the growth of ZnO NSs via ILE have led to successful growth of NSs from several other 2D metal oxides, including MnO_2_, Fe_3_O_4_, CoO, and Bi_2_O_3_.^[^ ^9^ ^]^ Our previous study has also revealed the significant influence of ionized amphiphilic monolayer on the nanosheet morphology.^[^ ^9^, ^10^, ^11^, ^12^ ^]^ With the assistance of the free‐standing charged surfactant template, metal ions concentrated and nucleated in the EDL zone resulting in 2D nanocrystals. While ILE is believed to be able to synthesize NSs from a broader range of materials, the generality is limited by the requirement of different hydrothermal reaction conditions. Particularly, heating required to activate the reaction often leads to additional nucleation formed in the bulk solution yield nanoparticles (NPs) byproducts. In this paper, we report that tartrate ligands could coordinate the metal precursor ions in the aqueous solution,^[^ ^13^, ^14^ ^]^ and could effectively prevent nucleation in the bulk solution phase and promote the precursor accumulation at the air‐water interface. This synthesis strategy allowed NSs growth in ambient conditions and broadened the synthesis of ultrathin NSs to a large range of single‐element or multi‐element metals, metal oxides, and hydroxides. We also leveraged a multimodal Large Language Model (LLM) to evaluate the quality of NS from easily obtained Scanning Electron Microscopy (SEM) images, enabling automated, rapid, consistent and unbiased assessment of sample quality. This capacity establishes a novel framework for the objective comparison of synthesis outcomes derived from diverse methodologies and research entities. The discovery of tartrate‐assisted ILE system, together with the machine learning (ML)‐based LLM methods, shows a path to a general and scalable synthetic approach for creating ultrathin 2D nanomaterials from the versatile families of functional materials in the field of laboratory automation.

## 2 Results and Discussion

### 2.1 Synthesis of NSs from Different Elements

**Figure 1:**
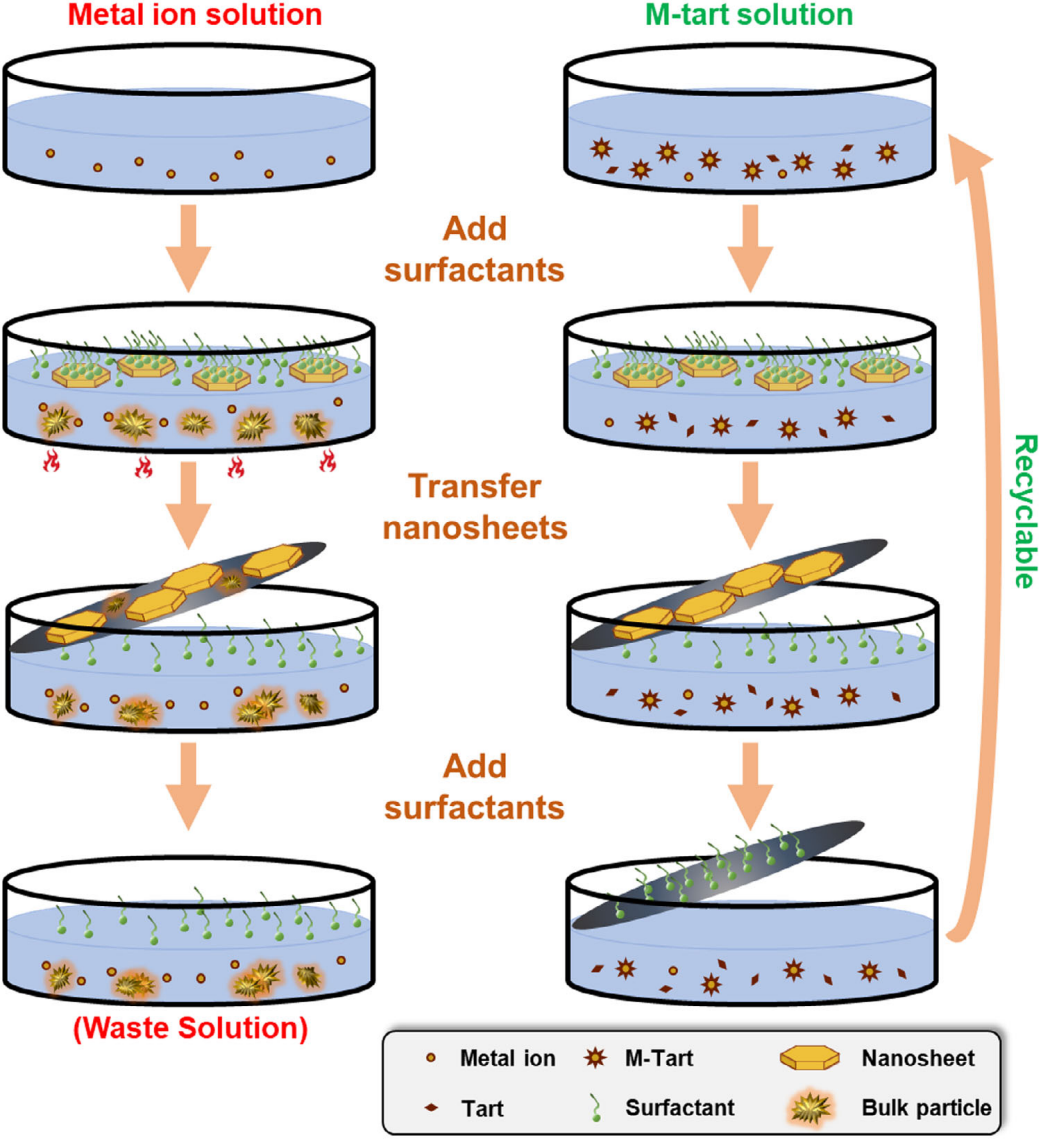
Schematic comparison of regular ILE (left column) and tartrate‐assisted ILE (right column) processes.

The difference between the tartrate‐assisted ILE and regular ILE synthetic processes is schematically shown in **Figure** **1**a (Synthesis details are in the Experimental Section). In a regular ILE process, an elevated temperature (e.g., 60 °C for ZnO) was required for hexamethylenetetramine decomposition to initialize the hydrolysis of metal ions. This affected both the EDL‐confined 2D interface and the bulk solution. As a result, the post‐synthesis solution contained NPs, which often attached to the NSs, becoming a major impurity after transferring affecting the final performance. Because tartrate can coordinate with metal ions in the solution,^[^ ^13^ ^]^ it thereby can stabilize the precursors and prevent the formation of NPs in the solution. Meanwhile, tartrate‐coordinated metal ions would show better affiliation to the surfactant monolayer, enabling nucleation and crystal growth at the room temperature. Because the precursors only particulate in 2D NSs growth at the air‐water interface, a substantial amount of precursors remain in the post‐synthesis solution, which can be recycled for another synthesis after removing the NSs products and surfactant residues at the interface.^[^ ^15^ ^]^

**Figure 2:**
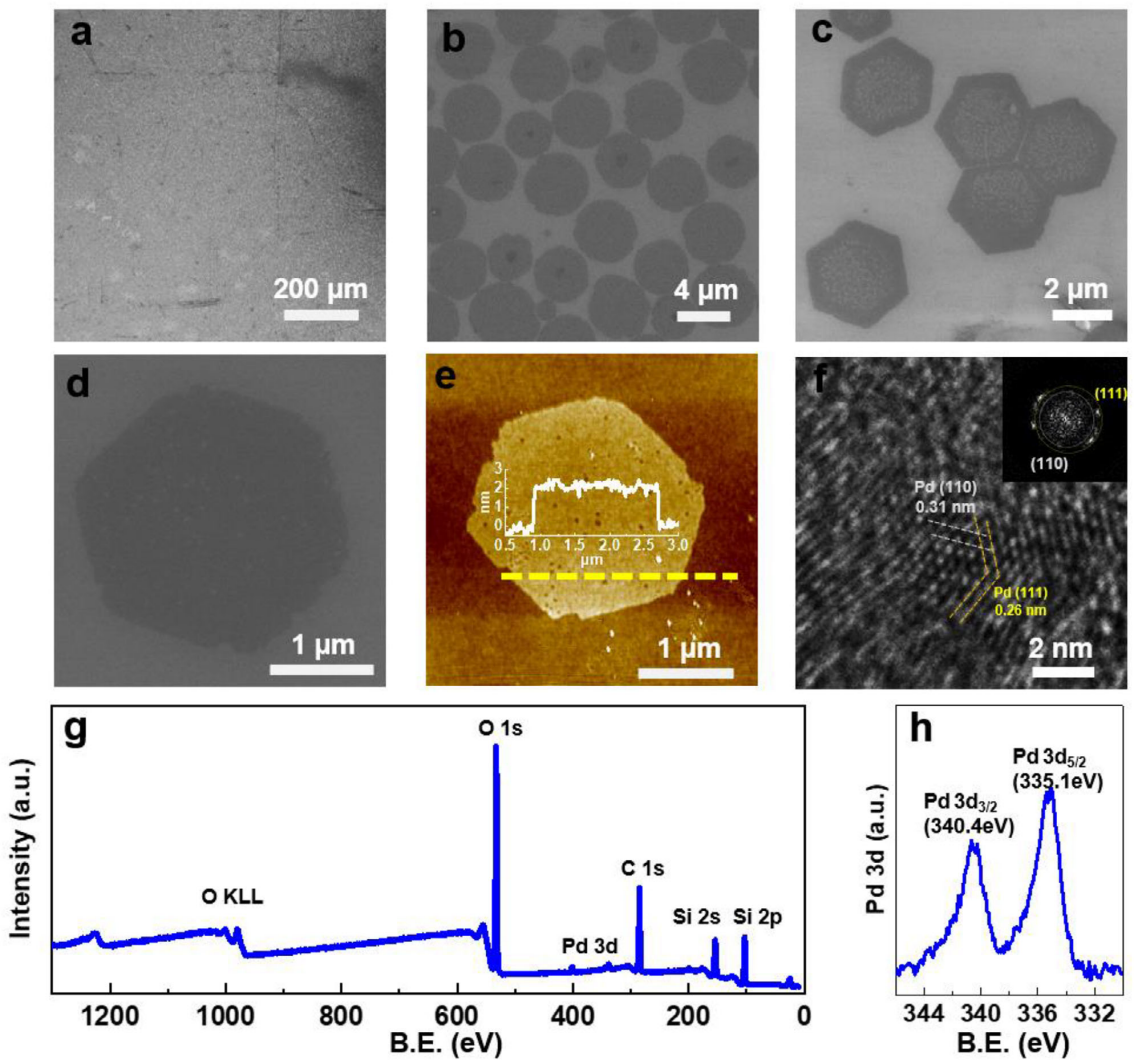
Pd NSs synthesized by tartrate‐assistant ILE. a) Low‐magnification SEM image showing the uniform coverage of Pd NSs on a Si substrate. b) Higher‐magnification SEM image revealing the slight shape and size variation of Pd NSs. c) SEM image demonstrating interactions between neighboring hexagonal NSs, leading to edge‐to‐edge connected assembly. d) SEM image of a well‐defined, isolated hexagonal Pd NS, providing a clear view of its distinct morphology. e) AFM image of a single hexagonal Pd NSs showing its uniform thickness, the inset line profile is indicated by the dashed line across the NS. f) HRTEM image of a Pd NS revealing the lattice matching well with the Pd (110) and (111) plane. Inset is the corresponding fast Fourier transform pattern (FFT) confirming its crystallographic orientation. g) XPS survey spectrum of Pd NSs, showing the elemental composition. h) XPS Pd 3d spectrum of Pd NSs, detailing the chemical states of palladium.

The tartrate‐assisted ILE process was first applied to the synthesis of Pd NSs, where amphiphilic octadecylamine (ODAM) and PdCl_2_ were used as the surfactant template and precursor, same as regular ILE.^[^ ^16^, ^17^ ^]^ Adding additional ammonium tartrate allowed the formation of Pd NSs at ambient condition in 3 h. The as‐grown NSs were transferred onto a SiO_2_‐coated conductive Si substrate surface by scooping from the air‐water interface. The as‐transferred NSs were densely packed without overlapping and covered nearly the entire surface (**Figure** **2**a; Figure S1, Supporting Information). Closer observation showed that the NS shapes and sizes were slightly irregular due to their compact distribution (Figure 2b). Various interactions were found between neighboring hexagonal NSs. As NSs get close to each other, they could rearrange their orientations and assemble into an edge‐to‐edge connected structure (Figure 2c). The boundary regions between NSs could be filled by an extended growth and yielded a merged NS over a larger size. This morphological evolution followed the oriented attachment.^[^ ^18^, ^19^ ^]^ In addition, no NPs were found at the air‐water interface and in the post‐synthesis solution (Figure S2, Supporting Information). This proved that tartrate could prevent the NPs formation and preserve the high quality of the precursor solution, suggesting the potential for reusing the post‐synthesis solution over time. The morphological evolution of these nanosheets was monitored through a time‐dependent synthesis, which confirmed a growth pathway from initial circular disks to faceted hexagons that later merge via oriented attachment. (Figure S3, Supporting Information)

Closer observation showed that the as‐grown Pd NS had a hexagonal shape with side lengths of 1.5–2.0 µm (Figure 2d). Atomic force microscopy (AFM) topographic image revealed that the as‐received NS had a flat surface with an average roughness of 0.19 nm and a uniform thickness of 2.1 nm (Figure 2e). It should be noted that any nanoparticle by‐products, if exist, would have distinct thickness from the ultrathin NSs, and would show clear contrast in SEM and observable height variation in AFM. Considering the large areas observed by these two techniques, the combination of SEM and AFM analyses provides reliable and consistent evidence of the clean and particle‐free NS surfaces. The crystal structure of the Pd NS was characterized by high‐resolution transmission electron microscopy (TEM) (Figure 2f). The d‐spacings of 0.31 and 0.26 nm could be indexed to the {110} and {111} facets, respectively, matching well to FCC Pd (JCPDS No.46‐1043).^[^ ^20^ ^]^ The corresponding fast Fourier transform (FFT) pattern confirmed that the crystalline domain had an exposed (110) surface plane, which has also been observed from Pd NSs synthesized by other approaches.^[^ ^21^, ^22^ ^]^ X‐ray photoelectron spectroscopy (XPS) was implemented to investigate the chemical characteristics of the Pd NSs (Figure 2g). A full wavelength survey scan of the NSs showed the Pd was the strongest signal other than the elements from the SiO_2_‐coated Si substrate. The high‐resolution Pd 3d spectrum confirmed the Pd^0^ oxidation state of the NSs (Figure 2h). The Pd 3d_5/2_ and Pd 3d_3/2_ peaks located at 335.1 and 340.4 eV, respectively, matching well with the characteristic binding energies of metallic Pd. The above characterizations evidenced the formation of 2D hexagonal Pd NSs from the air‐water interface. Because the extremely small amount of metal ions was converted to Pd NSs in each synthesis, it is believed that the synthetic process can maximize the precursor utilization and keep running with replenishing metal ions (Figure S4, Supporting Information).^[^ ^15^ ^]^

**Figure 3:**
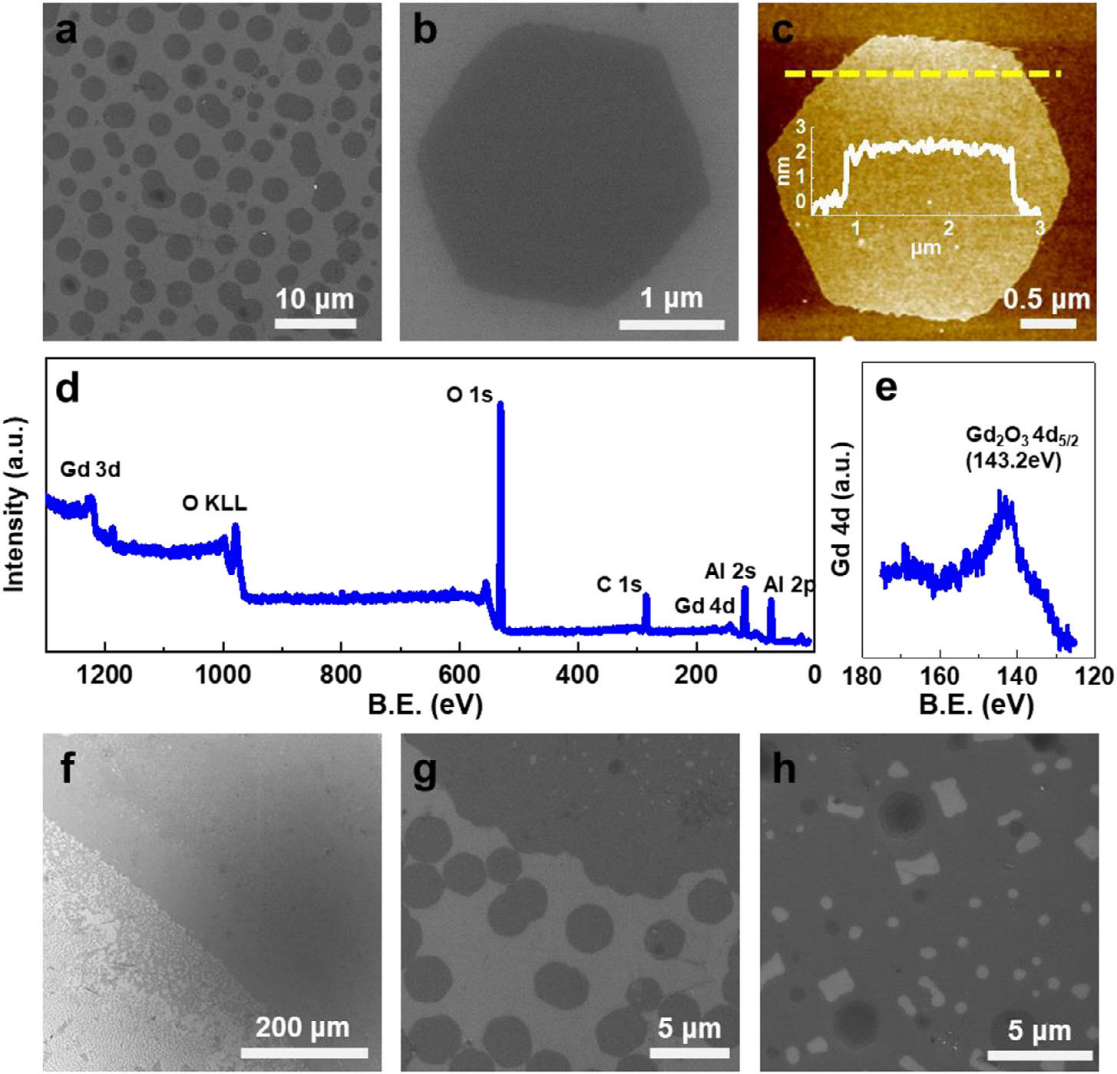
Gd_2_O_3_ NSs synthesized by tartrate‐assistant ILE. a) Low‐magnification SEM image showing the uniform distribution of hexagonal Gd_2_O_3_ NSs across the substrate. b) Higher‐magnification SEM image revealing the distinct hexagonal morphology of an individual Gd_2_O_3_ NSs. c) AFM image of a single hexagonal Gd_2_O_3_ NS, with an inset showing its thickness profile indicating a uniform and ultrathin morphology. d) XPS survey spectrum of Gd_2_O_3_ NSs, displaying the overall elemental composition. e) XPS Gd 4d spectrum of Gd_2_O_3_ NSs, showing the characteristic peak at 143.2 eV. f) Low‐magnification SEM image demonstrating wafer‐scale coverage of Gd_2_O_3_ NSs obtained from a 5 h synthesis. g) A SEM image showing Gd_2_O_3_ NSs assembled and packed densely at the edge of assembly area. h) An SEM image taking at the center of assembly area showing interconnect Gd_2_O_3_ NSs.

The tartrate‐assisted ILE was further applied to the synthesis of NSs from other elements, starting with the 5d rare earth elements. As the example shown in **Figure** **3**, Gd_2_O_3_ NSs were synthesized through the same approach by using Gd (NO_3_)_3_ as the precursor through the growth time of 2 h. The as‐received Gd_2_O_3_ NSs were densely distributed at the air‐water interface and readily transferred to a substrate (Figure 3a). They also exhibited a hexagonal structure with edge lengths of 1–2 µm (Figure 3b) and a uniform thickness of 2.2 nm (Figure 3c). This sharp‐edged hexagonal morphology is a strong indicator of high crystallinity, which is a correlation we previously established from a number of other NSs by ILE using synchrotron grazing incidence X‐ray diffraction (GIXRD).^[^ ^10^ ^]^ XPS was carried out with Gd_2_O_3_ NSs on a sapphire substrate (Figure 3d). The binding energy of the Gd 4d_5/2_ was 143.2 eV associated with the oxidation state of Gd^3+^ in Gd_2_O_3_ (Figure 3e). Gd_2_O_3_ NSs showed a fast‐merging behavior, where a wafer‐scale continuous NS was obtained after 5 h synthesis. Some bridge connections were observed between the assembled Gd_2_O_3_ NSs (Figure 3f–h). The precursor solution could also be reused for at least 5 synthesis cycles without noticeable degradation (Figures S5 and S6, Supporting Information).

**Figure 4:**
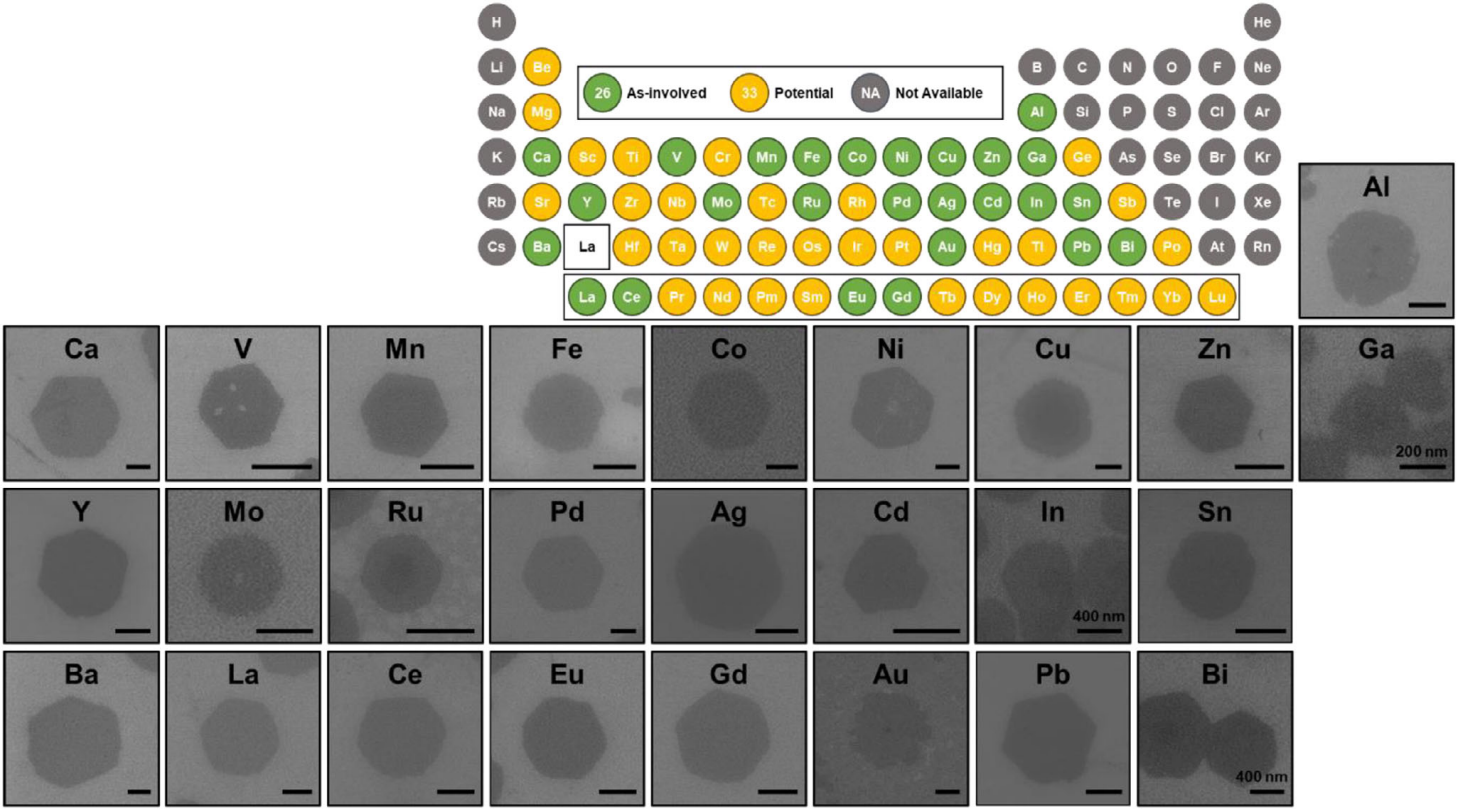
Summary of tartrate‐assisted ILE synthesis of 2D NSs from all possible elements. Top: A periodic table showing the single elements that can be synthesized into NSs by tartrate‐assisted ILE. Green (As‐involved): elements that have been successfully synthesized into NSs. Blue (Potential): Elements that have potential to be synthesized into NSs. Gray (Not Available): Elements that may not be feasible to be synthesized into NSs. Bottom: A collection of representative SEM images showing the morphology of single‐element NSs synthesized from all possible elements (scalebar = 1 µm).

The tartrate‐assisted ILE synthesis was then applied to all elements which have available water‐soluble precursors (**Figure** **4**, elements without available precursors were marked with gray). Micrometer‐sized hexagonal single‐element NSs were successfully obtained from 26 metal elements (elements marked with yellow circle). Representative SEM images of individual NSs from each successfully synthesized element are shown together with the periodic table in Figure 4. All of them exhibited isolated NSs morphology with a hexagonal shape. Their thickness was also ≈1 to 2 nm. The detailed synthesis parameters are summarized in Table S1 (Supporting Information). According to these parameters, we discovered some trends across the periodic table. First, lighter elements generally required higher concentration, lower ODAM density, and longer growth time to form a regular hexagonal shape. Second, the metal ion to tartrate molecule ratio reduced as the atomic number increases. Third, the merging phenomenon was stronger in 3d transition metal oxide NSs, such as CoO, Co (OH)_2_ (Figure S7, Supporting Information). A nontrivial “tiger‐skin” like surface feature was found on their merged NSs (Figure S8, Supporting Information). These results demonstrated the excellent versatility and universality of the tartrate‐assisted ILE methodology and revealed its potential of it to be applied for synthesizing ultrathin 2D NSs from a myriad of elements.

**Figure 5:**
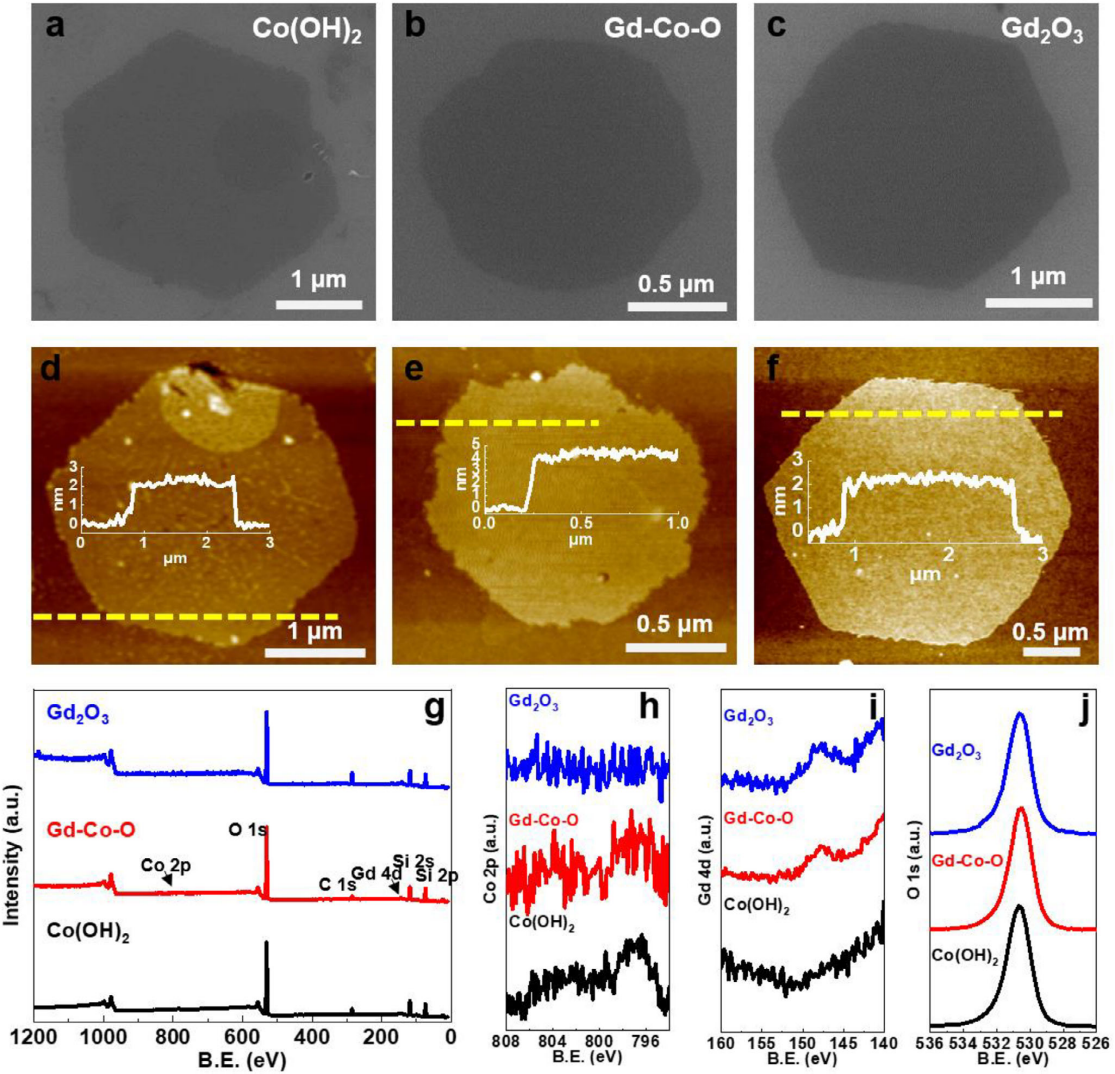
Gd‐Co‐O NSs synthesized by tartrate‐assistant ILE. SEM images a–c) and AFM topographies d–f) of Co (OH)_2_ NSs, Gd‐Co‐O NSs, and Gd_2_O_3_ NSs, respectively. g) Survey XPS spectra of Co (OH)_2_, Gd‐Co‐O, and Gd_2_O_3_ NSs. h–j) Enlarged XPS spectra showing the peak regions of Co 2p h), Gd 4d i), and O 1s j).

Beyond the single‐element NSs, the ILE method could also be used to synthesize multi‐element and are promising for creating so‐called high‐entropy or compositionally complex NSs.^[^ ^23^, ^24^ ^]^ As an example, Gd‐Co‐O NSs were obtained by using a mixed precursor solution of CoCl_2_ and Gd(NO_3_)_3_ in the presence of tartrate. A 3 h growth yielded hexagonal Gd‐Co‐O NSs that showed similar size and distribution as those obtained from single‐element NSs (Figure S9, Supporting Information). Closer comparation of individual NSs revealed that the Gd‐Co‐O NSs had relatively rougher edges compared with the single‐phase Co (OH)_2_ and Gd_2_O_3_ NSs (**Figure** **5**a–c). A flat and uniform surface was observed from Gd‐Co‐O NS, The Ra from the AFM data of Co(OH)_2_, Gd‐Co‐O, and Gd_2_O_3_ NSs are 0.18, 0.17, and 0.21 nm, respectively. All these exhibit excellent 2D planar smooth features, evidencing the growth results from Gd‐Co‐O were comparable to those of single‐phase NSs (Figure 5d–f). However, the thickness of Gd‐Co‐O NS was found to be ≈4.4 nm, which was double of the single‐phase NSs. XPS analysis confirmed the existence of both Co and Gd in the Gd‐Co‐O NSs (Figure 5g). The Co 2p spectrum of Co(OH)_2_ displayed characteristic peaks at ≈781 and 797 eV, corresponding to Co 2p_3/2_ and Co 2p_1/2_, respectively. In addition, prominent shake‐up satellite features were observed, which were indicative of Co^2+^ in a high‐spin configuration. In the Gd‐Co‐O NSs, the Co 2p region demonstrated a slight shift to higher binding energy and a broadening of the peaks in comparison to Co(OH)_2_, indicating a modification in the chemical environment of Co due to its interaction with Gd and O. The presence of these features is consistent with a mixed‐valence state or the coexistence of Co^2+^ and Co^3+^ species, which may be attributable to surface oxidation or interaction with Gd (Figure 5h). The Gd 4d region exhibited peaks at 142 and 148 eV, which are consistent with Gd^3+^ in Gd_2_O_3_. A slight shift in the Gd 4d peaks of the Gd‐Co‐O NSs compared with Gd_2_O_3_ may be attributed to the interaction of Gd with Co and O, indicating a change in the electronic environment of Gd (Figure 5i). The O 1s spectrum of Co (OH)_2_ exhibits a peak centered ≈531 eV, indicative of hydroxide (OH^−^) species. In Gd_2_O_3_, the O 1s peak was observed at a lower binding energy (≈529 eV), characteristic of metal‐oxide (O^2−^) bonds. The Gd‐Co‐O NSs demonstrated a more extensive O 1s peak, which may be attributed to the presence of both oxide and hydroxide species, potentially bridging oxygen at the Gd‐O‐Co interface. This broadening of the peak suggests the existence of multiple oxygen environments (Figure 5j). The uniform morphology observed via microscopy, coupled with XPS data indicating atomic‐level interactions between the constituent elements, suggests a homogeneous elemental distribution within the multi‐element nanosheets.

In another demonstration, the tartrate‐assisted ILE was also applied to the synthesis of Co‐Ni bi‐element NSs by using CoCl_2_ and NiCl_2_ as the precursors. Because both Co and Ni are 3d transition metals and their chemical properties are very close, the mixed precursor solution could yield a single‐phase bi‐element Co_x_Ni_1‐x_(OH)_2_ NSs structure. The morphology of Co_x_Ni_1‐x_(OH)_2_ NSs possessed no differences compared with Co(OH)_2_ and Ni (OH)_2_ NSs (Figure S10, Supporting Information). The oxidation states of Co and Ni in Co_x_Ni_1‐x_(OH)_2_ NSs exhibited the same binding energies as that of their single‐element NSs, and the intensities of Co and Ni peaks in Co_x_Ni_1‐x_(OH)_2_ NSs agreed with concentration ratio between Co and Ni (2:8 in this case) in the solution. With deeper understanding of the growth kinetics of single‐element NS, more complex 2D systems with controlled element distribution could be achieved.

### 2.2 Electrochemical Property of NSs

**Figure 6:**
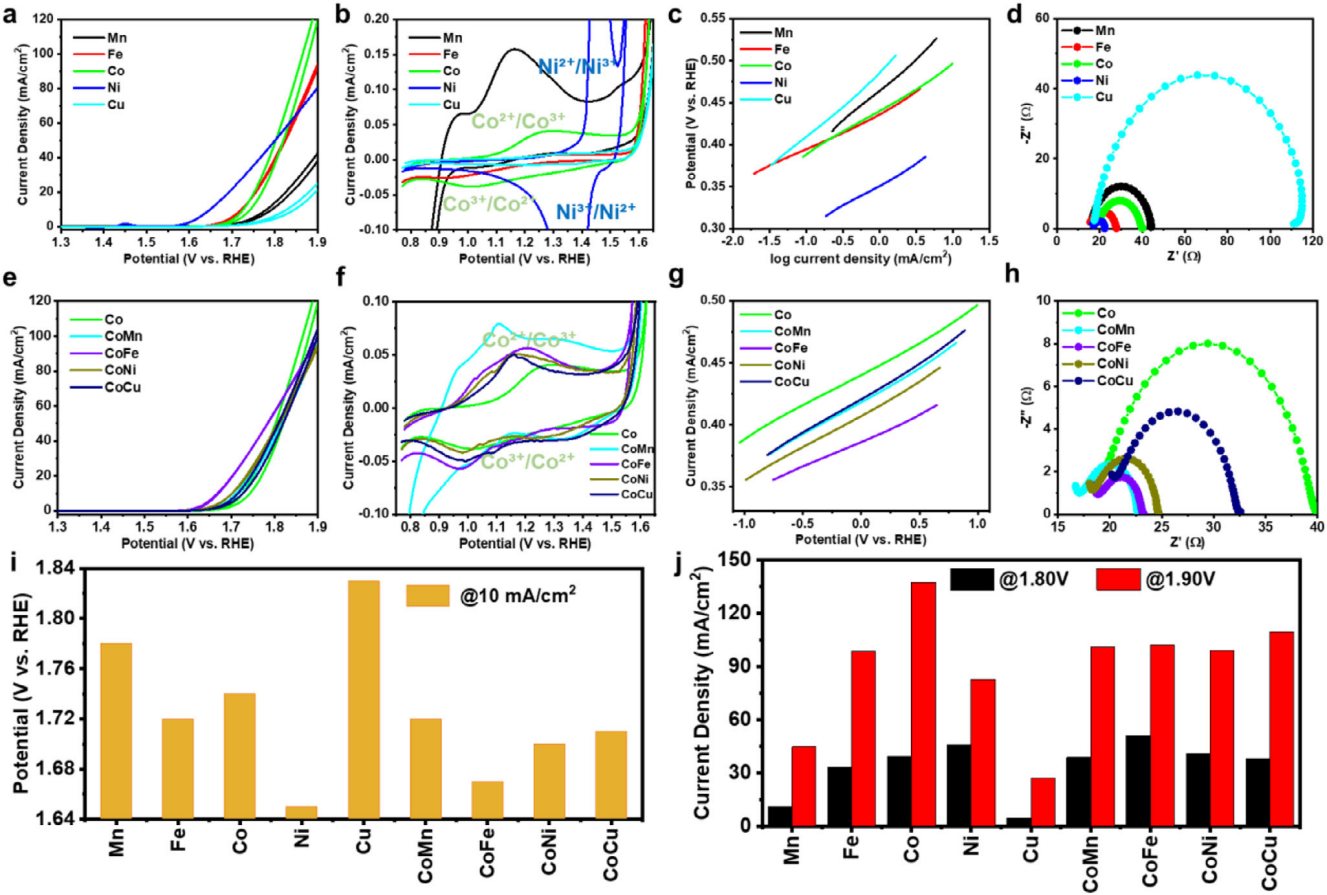
Electrochemical OER performance comparison of NSs. a–d) OER performance of the single‐element NSs: a) LSVs. b) Cyclic voltammograms (CVs) showing characteristic redox peaks with key transitions (Co^2+^/Co^3+^, Ni^2+^/Ni^3+^) indicated. c) Tafel slopes. d) Nyquist plots. e–h) OER performance of the multi‐element NSs: e) LSVs. f) CVs highlighting the characteristic Co^2+^/Co^3+^ redox features. g) Tafel slopes. h) Nyquist plots. i) Comparison of the OER potentials at the current density of 10 mA cm^2^ for all NSs. j) Current densities at fixed potentials of all NSs.

A comparative study of single‐element (Mn, Fe, Co, Ni, Cu, as shown in **Figure** **6**a–d) and multi‐element nanosheets (NSs, as shown in Figure 6e,f) was conducted to reveal a complex relationship between morphology, composition, and property (measured by the oxygen evolution reaction (OER) electrochemical activity).^[^ ^25^ ^]^ The OER characterization was conducted at 25 °C in an alkaline (1 M KOH) electrolyte with a pH of 14 (see experimental section for details). Among single‐element NSs, Ni‐based NSs, featuring a well‐defined, sharp hexagonal morphology (Figure S11a, Supporting Information), demonstrate the best OER performance as evidenced by their lowest overpotential in Linear Sweep Voltammetry (LSV) (Figure 6a), where the current density increased significantly at a lower applied potential compared to other materials, indicating superior catalytic activity. It also exhibits a high Electrochemically Active Surface Area (ECSA) as revealed by its larger capacitive current in the Cyclic Voltammetry (CV) analysis (Figure 6b), indicating there might be a large number of active sites on Ni NSs. The cyclic voltammograms (Figure 6b,f) reveal characteristic redox peaks for the Co‐ and Ni‐based catalysts. The anodic peak observed prior to the onset of the OER corresponds to the oxidation of the metal hydroxide to the metal oxyhydroxide (e.g., Co(OH)_2_ to CoOOH), which forms the active catalytic sites for the reaction.^[^ ^15^, ^17^ ^]^ Furthermore, the Ni‐based NSs show the fastest reaction kinetics, as indicated by their lowest Tafel slope (Figure 6c), revealing a highly efficient and favorable reaction pathway for the OER. From Electrochemical Impedance Spectroscopy (EIS) (Figure 6d), the Ni‐based NSs also displayed the smallest semicircle in the Nyquist plot, suggesting the most efficient charge transfer at the electrode‐electrolyte interface. This excellent performance could be attributable to a higher density of active sites, a favorable electronic structure with minimal defects or trapping sites, and improved mass transport. As the NS morphology became more defective with a less‐defined shape, the OER performance decreased following the order of Ni > Co > Fe > Mn > Cu. This relationship was further investigated from multi‐element NSs (80% Co combined with 20% of Mn, Fe, Ni, or Cu) (Figure 6e–h). While these multi‐element NSs exhibited a less distinctly hexagonal shape (Figure S11c, Supporting Information) than the single‐element NSs (Figure S11a, Supporting Information), the electrochemical data revealed a striking enhancement in their OER performance (Figure 6e–h). Among them, the Co‐Fe NSs exhibited superb electrochemical activity surpassing all the other bi‐ and single‐element NSs. This finding indicates that the synergistic electronic interaction between dislike elements, such as Co and Fe plays another critical role in controlling the NS's OER activity.

The potentials at a standard current density (10 mA cm^2^) (Figure 6i) and current densities at fixed potentials (Figure 6j) of all NSs were summarized and compared together. The data shows that Ni, Co, CoNi, and CoFe nanosheets exhibit superior OER performance, requiring significantly lower overpotentials and achieving substantially higher current densities compared to the other materials tested. Specifically, our ultrathin Ni NSs require a potential of ≈1.65 V versus reversible hydrogen electrode (RHE) to reach 10 mA cm^2^ (Figure 6i), a performance that is highly competitive with Ni(OH)_2_ catalysts prepared by conventional methods such as electrodeposition, which needs ≈1.67 V at a similar condition.^[^ ^18^ ^]^ This comparison suggests our tartrate‐assisted ILE synthesis was able to provide quality NSs with comparable functionality as those synthesized by conventional ILE. The data strongly suggest that Co is a highly active component for OER, and that its activity can be further enhanced by combining with Ni and Fe. These findings are consistent with literature reports on Ni, Co, and CoFe OER catalysts.^[^ ^17^, ^26^ ^]^ The enhanced activity observed in CoNi and CoFe catalysts may be attributed to modifications in their electronic structure, the emergence of novel active sites at the interfaces, or alterations in the surface adsorption properties of OER intermediates.^[^ ^27^ ^]^

### 2.3 Multimodal Large Language Models for NS Analysis

With the potential to achieve a large number of elemental combinations through this high‐throughput synthesis method, it is also essential to have a compatible assessment strategy to evaluate the quality of these synthesized NSs. Here, we employed the vision capabilities of multimodal large language models (LLM) to assess the quality of 2D nanosheets, demonstrating a pathway toward high‐throughput and standardized evaluation of ILE synthesis results. The use of multimodal LLMs is a new strategy that can analyze various forms of information, including images.^[^ ^28^ ^]^ By harnessing the ability of multimodal LLMs to interpret images in the context of detailed textual descriptions, we aim to develop a system that not only matches but surpasses traditional methods in flexibility, efficiency, and reproducibility. Our study focused on assessing the model's performance by applying subjective scoring criteria accurately and consistently across diverse samples, thereby determining its viability as an AI‐assisted tool for automatic evaluation of the quality of NSs.^[^ ^29^, ^30^ ^]^

To develop our LLM‐based approach, 36 SEM images of NSs grown under various conditions were selected, representing a wide range of morphological qualities. The contrast and sharpness of all the images were normalized and all the labels were removed to ensure fair assessments (Figures S12–S14, Supporting Information). The key metric used to assess the consistency of scoring was the intraclass correlation coefficient (ICC), which measures the correlation between different sets of scores, allowing for comparing the consistency across any number of scorers (Supporting Material Methods section). To establish a baseline, the scoring was first conducted by six human experts, who have extensive experience in the growth and characterization of nanostructures. The quality of NS was scored based on a set of specific criteria, including edge straightness and equality, angles between each edge, corner sharpness, and structure symmetry for individual NSs, as well as the morphology consistency, uniformity, and separation across the sample.

To establish a consistency benchmark in our standard setup, representative examples were selected from the synthesized NSs. Specifically, for each of the elemental compositions (Ni, Bi, Ce, Co, La), we chose three representative images demonstrating poor, medium and good NS quality. The corresponding syntheses conditions are included in Table S2 (Supporting Information). The resulting ICC (3) was 79%, which was considered somewhat less than ideal. To assess interscorer consistency, one expert scored the same image again after a two‐week interval. The ICC (3) for repeated scoring by the same individual was 90%, which is reasonably good, but still not very impressive considering it is a measure of consistency between the same person on the same data just two weeks apart. Although the number of scorers and samples limits a fully quantitative assessment, a solid qualitative conclusion can be drawn. There was considerable variation in the scores assigned by different experts, even when using explicit criteria and having received the same training. Furthermore, individual scorers showed non‐trivial variability when scoring the same set of images multiple times. This emphasizes the need for a quick, consistent, and unbiased automated scoring system.

**Figure 7:**
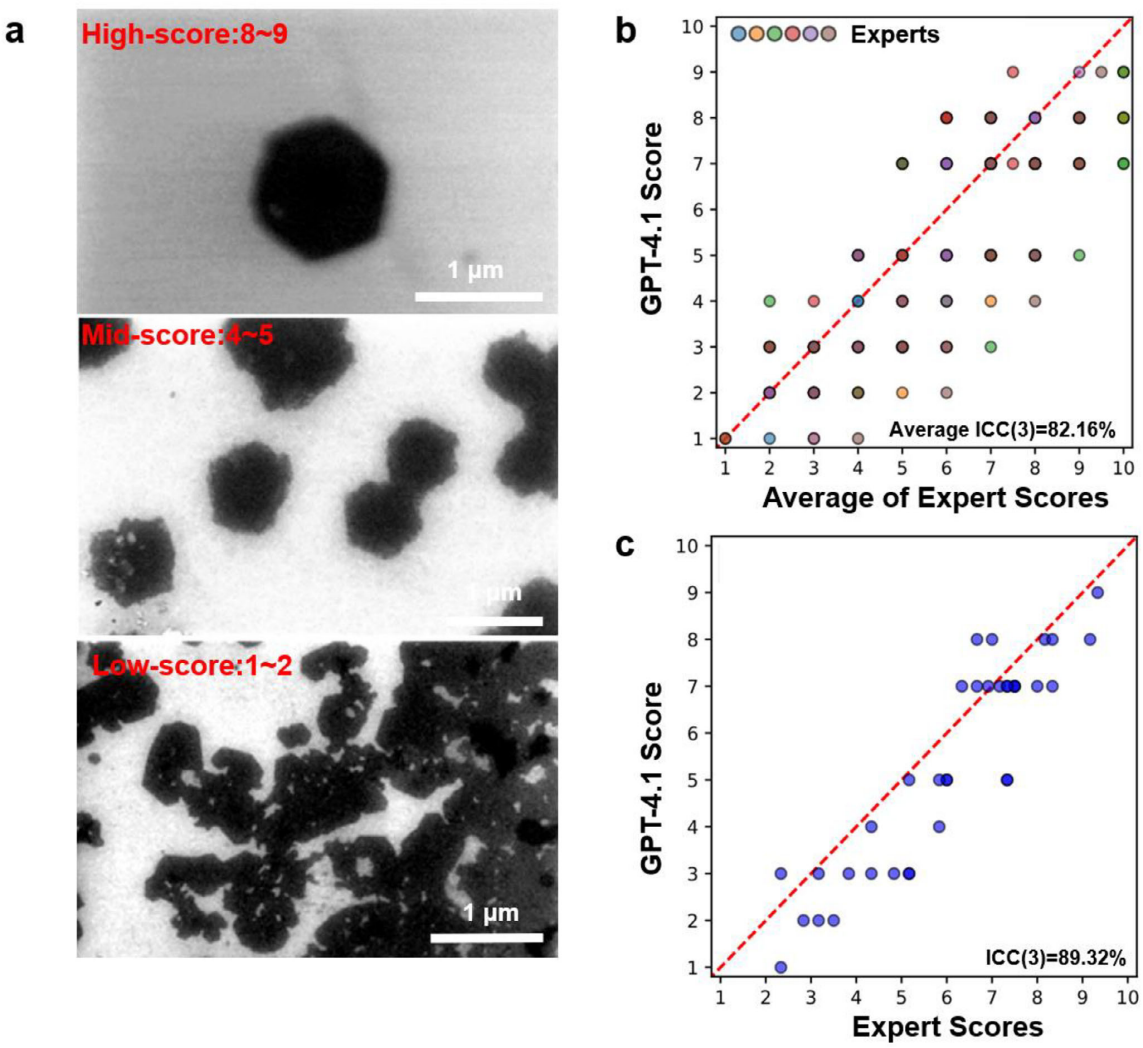
LLM model for NS quality evaluation. a) Example images of three quality levels of as‐synthesized NSs. b) Parity plots for image scores of each expert (different colored points) compared to GPT‐4.1 scores. Each dot represents an individual image evaluated by both a human expert and GPT‐4.1. The dashed line represents perfect agreement between expert scores and GPT‐4.1 scores. c) Average of expert scores compared to GPT‐4.1.

To test whether a multimodal LLM can serve as an automated scorer, we provided the model with the same set of information as the human scorers by utilizing a few‐shot approach. We first showed GPT‐4.1 the three example images (**Figure** **7**a) and a prompt outlining the scoring criteria (see Supporting Information) and then asked the LLM to score the whole set of 36 images. The scores generated by GPT‐4.1 were first compared to each of the experts separately, the combined result of which is present in Figure 7b, with differently colored points representing scores from different experts. This comparison resulted in an average ICC (3) of 82%, similar to the ICC (3) of all human experts. This indicates that GPT‐4.1 in its own scoring behaves almost exactly as another independent expert scorer. Then, the GPT‐4.1 scores were compared to a likely more accurate evaluation of the images – the average score of human experts. As shown in Figure 7c, a good correlation value of ICC (3) of 89% was achieved, better than the ICC between human expert scorers and as good as the ICC between the same scorer two weeks apart. The average coefficient for each human versus the average of the other human experts was 87% +/‐ 5 pp, somewhat worse than the LLM. This suggests that GPT‐4.1 performed at least as well as a human expert and perhaps better, as it achieved a stronger correlation with the likely superior average than each person did. However, unlike human scorers, GPT‐4.1 provides consistent results, is readily available, always consistent, and can rapidly score thousands of images. This strong correlation, with an ICC value of 89% against the averaged expert scores, validates the LLM as a robust tool for high‐throughput quality assessment, capable of achieving a level of consistency and objectivity comparable to that of human experts.

## 3 Conclusion

In summary, a versatile tartrate‐assisted ILE methodology was developed for the synthesis of ultrathin 2D NSs in ambient conditions. Tartrate stabilization of metal ions prevents bulk nucleation while promoting precursor accumulation within the EDL, enabling rapid formation 2D NSs at the air‐water interface and elimination of impurities. As a result, this methodology demonstrates successful NS growth from 26 different elements under very similar growth conditions. This synthesis method was also applied to several multi‐elements systems, showing the potential for creating high‐entropy NSs. Most growth results exhibited high NS coverage with uniform nanometer‐scale thicknesses and smooth surfaces. Electrochemical characterizations were conducted on the transferred 2D NSs, and provided a comprehensive comparison of the OER performance against morphology and element combinations. A multimodal LLM method was implemented to provide autonomous quality assessment of the NSs. This LLM method, even without fine‐tuning or retraining, can effectively evaluate the quality of NSs, delivering consistent and unbiased assessments almost instantly. The optimized tartrate‐assisted ILE system with the LLM method promises a high‐throughput system for autonomously synthesizing and quality assessing 2D NSs throughout the entire periodic table, eventually enabling the discovery of 2D materials with unconventional elemental combinations of advanced catalysis design and beyond.

## Conflict of Interest

The authors declare no conflict of interest.

## Supporting information



Supporting Information

## Data Availability

The data that support the findings of this study are available from the corresponding author upon reasonable request.
