# Supplementary material for: Tartrate‐Assisted Ionic Layer Epitaxy for General Synthesis of 2D Nanostructures
Source: Small Methods. 2025 Nov 2;9(12):e01507. doi: 10.1002/smtd.202501507 (PMC12716201; doi:10.1002/smtd.202501507)
Supplement: Supplementary file 1 — Supporting Information [file SMTD-9-e01507-s001.pdf]

Supporting Information

Tartrate-Assisted Ionic Layer Epitaxy Synthesis of Two-dimensional Nanostructures at the Air-Water Interface

Ziyi Zhang, Derui Wang, Maciej P. Polak, Corey Carlos, Yutao Dong, Dane Morgan, Xudong Wang**

Department of Materials Science and Engineering, University of Wisconsin-Madison, Madison, Wisconsin 53706, United States.

Keywords: 2D materials, metal tartrate, ionic layer epitaxy, general synthetic method, high-throughput synthesis.

Materials and Experimental Methods

Synthesis of Pd NSs. The discrete Pd NSs were synthesized by using the ILE method. A 10 mL aqueous solution containing 0.02 mM Palladium (II) Chloride ($PdCl_2$) and 2 mM Tartrate (Tart) was prepared in a 20 mL glass vial (with an inner diameter of 24 mm) by subsequently dissolving ammonium L-tartrate (Sigma-Aldrich) and $PdCl_2$ (Sigma-Aldrich) powders. Then, 6 μL of a chloroform solution containing 1.8 mM octadecyl amine (ODAM, Sigma-Aldrich) was slowly added onto the precursor solution surface. Finally, this glass vial was kept in ambient conditions for 3 hours. The Pd NSs could be transferred onto an arbitrary substrate by scooping at the surface of the solution or extracting the solution from the vial bottom for characterization and device fabrication. Si substrates were used for NS characterizations. To synthesize the merged Pd NSs, 8 μL ODAM solution was used, and the synthesis time was elongated to 5 hours.

Synthesis of Gd_2O_3 NSs. The discrete Gd_2O_3 NSs was synthesized by using the ILE method. a 10 mL aqueous solution containing 0.02 mM Gadolinium (III) nitrate ($Gd(NO_3)_3$) and 2 mM Tartrate (Tart) was prepared in a 20 mL glass vial (with an inner diameter of 24 mm) by subsequently dissolving ammonium L-tartrate (Sigma-Aldrich) and $Gd(NO_3)_3 \cdot 6H_2O$ (Sigma-Aldrich) powders. Then, 8 µL of a chloroform solution containing 1.8 mM octadecyl amine (ODAM, Sigma-Aldrich) was slowly added onto the precursor solution surface. Finally, this glass vial was kept in ambient condition for 2 hours. To synthesize the merged Gd_2O_3 NSs, the synthesis time was elongated to 5 hours.

Materials Characterizations. A Zeiss LEO 1530 field-emission scanning electron microscope (FESEM) was used to study the morphologies of the NSs. Atomic force microscopy (AFM) tomography images were obtained using an XE-70 Park System. X-ray photoelectron spectroscopy (XPS) spectrum was obtained from a Thermo Scientific K-alpha XPS instrument at a 400 µm spot size, with the flood gun turned on during the measurements. An FEI TF30 transmission electron microscope operated at 300 kV was used to study the crystal structure of the samples.

Electrochemical Measurements

Electrochemical characterizations were performed using an Autolab PGSTAT302N potentiostat in a standard three-electrode configuration. Synthesized nanosheets on a fluorine-doped tin oxide (FTO) substrate (1 cm^2 exposed area) served as the working electrode, with a platinum wire as the counter electrode and a saturated calomel electrode (SCE) as the reference. All measurements were conducted in a 1 M KOH electrolyte. Potentials were converted to the Reversible Hydrogen Electrode (RHE) scale using the Nernst equation:

$$ERHE=ESCE+0.059\times pH+0.241\ V$$

Catalytic Performance Evaluation. The Oxygen Evolution Reaction (OER) activity was assessed via linear sweep voltammetry (LSV) at a scan rate of 50 mV/s. Tafel slopes, derived from the LSV data, were used to evaluate reaction kinetics. Electrochemical Impedance Spectroscopy (EIS) was performed at a constant overpotential to analyze charge transfer resistance at the catalyst-electrolyte interface. Long-term stability was evaluated through chronoamperometry (CA), where the current density was monitored over a 24-hour period at a fixed potential.

Electrochemically Active Surface Area (ECSA) Determination. The ECSA was determined by measuring double-layer capacitance (Cdl). This was achieved by performing cyclic voltammetry (CV) scans at multiple rates (e.g., 10, 20, 30, 40, and 50 mV/s) in a potential window where no Faradaic reactions occurred. The Cdl was calculated from the slope of a linear plot of the capacitive current density difference (Δj=ja−jc) against the scan rate. The ECSA was then calculated using the equation:

$$ECSA=Cdl/Cs$$

where Cs is the specific capacitance for a flat surface, assumed to be 0.040 mF/cm² in 1 M KOH.

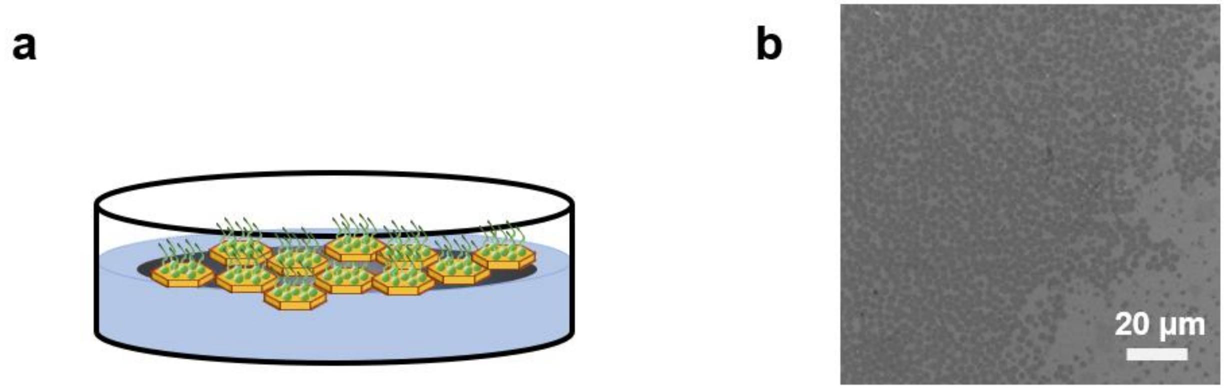

Figure S1. (a) Schematic of NS dense coverage on substrate. (b) SEM image-Pd overlap uniformly covers the surface.

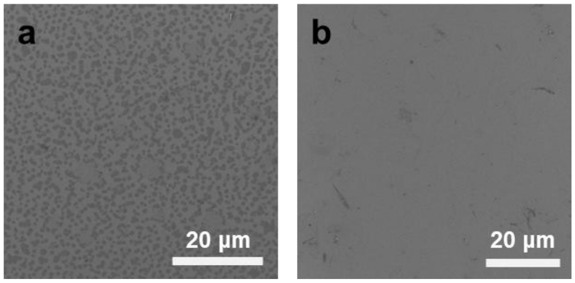

Figure S2. (a) SEM images of Pd NSs on $SiO2_2$-coated Si substrate. **(b)** SEM image of components from the post-synthesis solution.

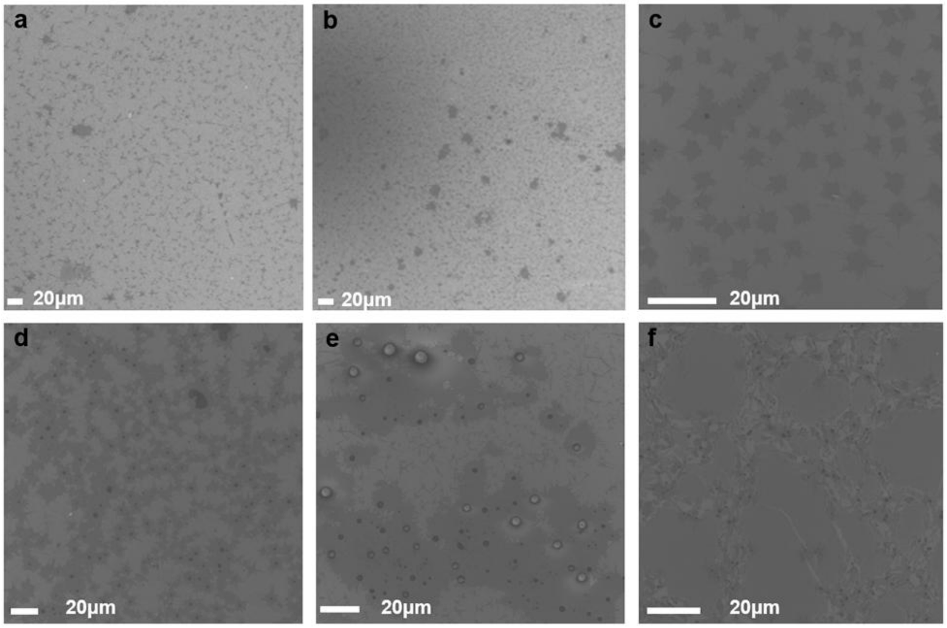

Figure S3. SEM images of Pd NSs obtained from a time-dependent synthesis, showing the morphological evolution at **(a)** 30 min, **(b)** 1 hr, **(c)** 2 hr, **(d)** 4 hr, **(e)** 6 hr, and **(f)** 24 hr .

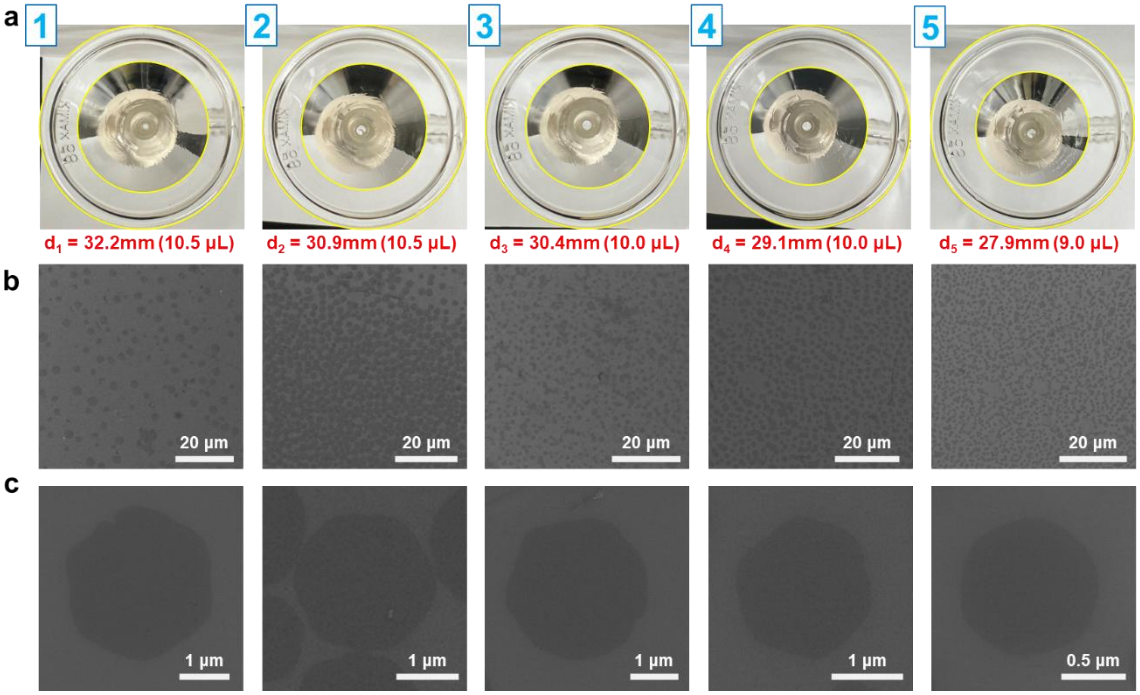

Figure S4. Pd NSs morphologies in the cycling synthesis. (a) Optical images and **(b-c)** SEM images of Pd NSs in repeated 3-hour synthesis. [Initial precursor solution: 10mL aqueous solution containing 0.02mM $PdCl_2$ and 2mM $(NH_4)_2$Tartrate.]

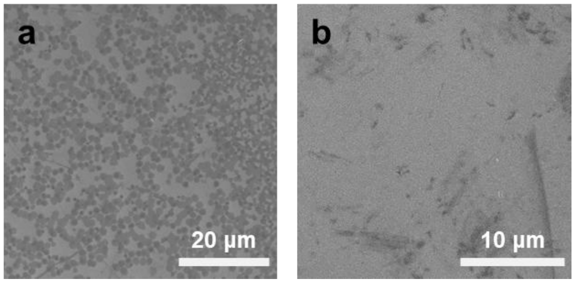

Figure S5. (a) SEM images of Gd_2O_3 NSs on $SiO2_2$-coated Si substrate. **(b)** SEM image of components from the post-synthesis solution.

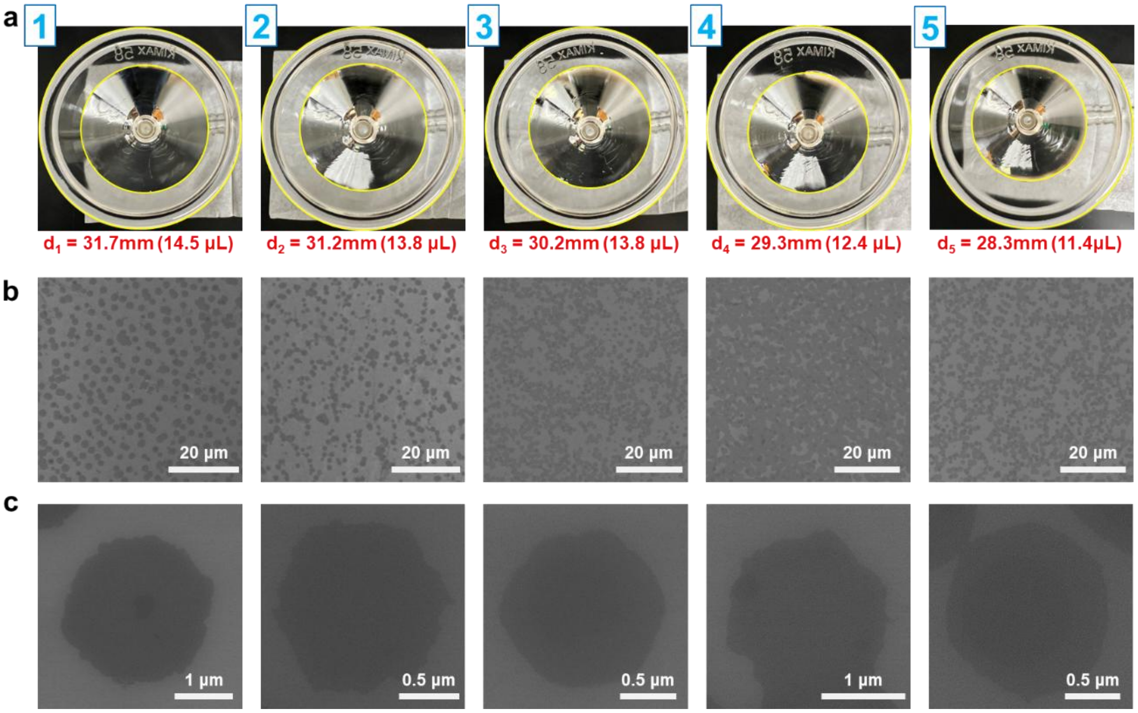

Figure S6. Gd_2O_3 NSs morphologies in the cycling synthesis. (a) Optical images and **(b-c)** SEM images of Gd_2O_3 NSs in repeated 2-hour synthesis. [Initial precursor solution: 10mL aqueous solution containing 0.02mM Gd $(NO_3)_3$ and 2mM $(NH_4)_2$Tartrate.]

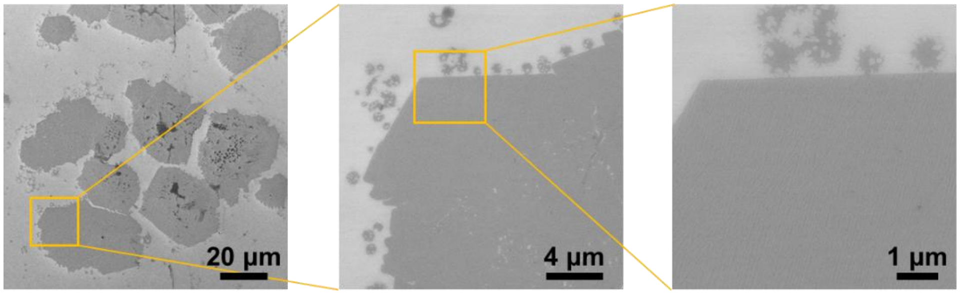

Figure S7. Ostwald Ripening & Oriented Attachment in the Co $(OH)_2$ NS structure evolution. SEM images at different magnifications of Co $(OH)_2$ NSs illustrate the ripening process.

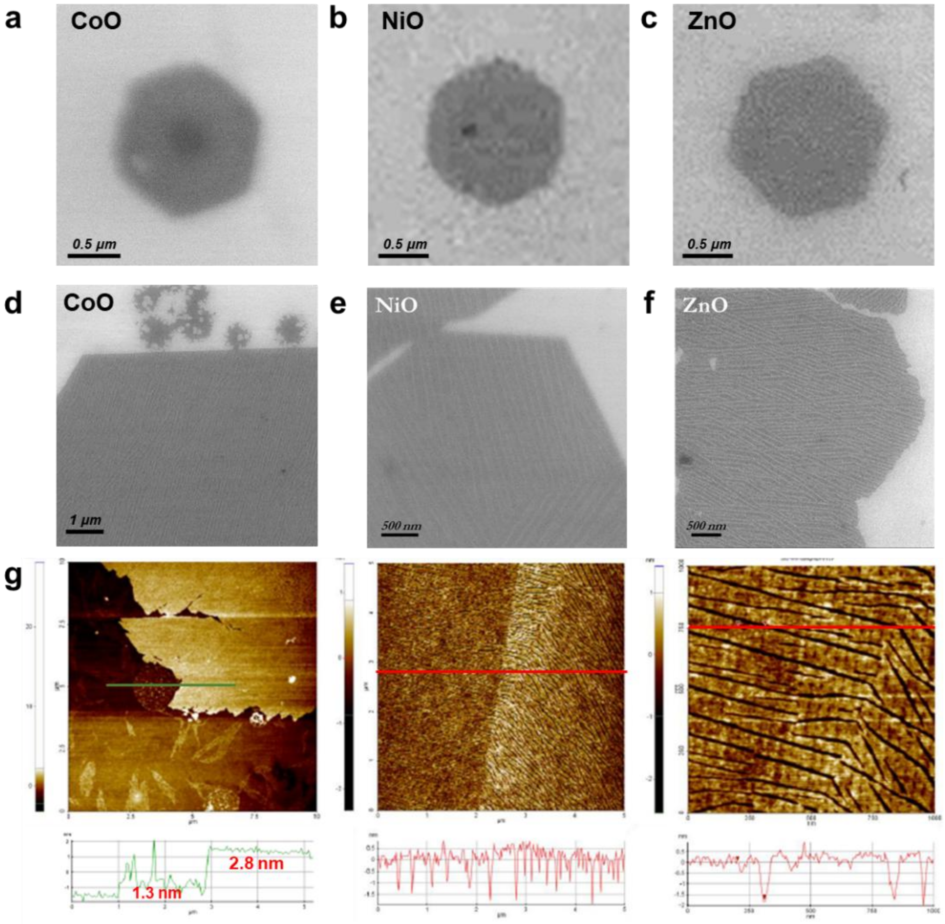

Figure S8. 3d transition metal oxide NSs from tartrate-assisted ILE growth. (a-c) Individual CoO, NiO and ZnO Hexagonal NSs from 1-hour synthesis. **(d-f)** Merged CoO, NiO and ZnO NSs from 4-hour synthesis. **(g)** AFM images of a merged NiO NS, showing the special "tiger skin" surface features.

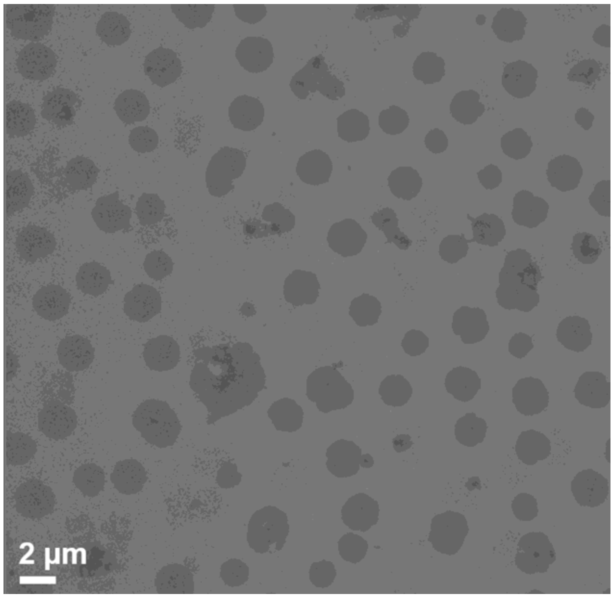

Figure S9. Gd-Co-O Alloy NSs from tartrate-assisted ILE growth.

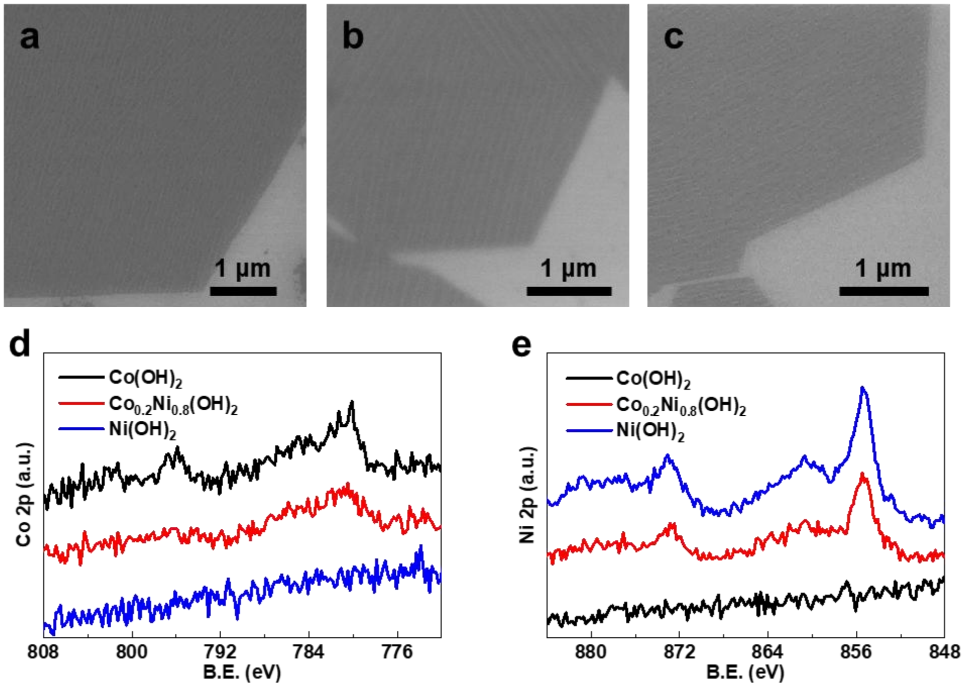

Figure S10. SEM images of **(a)** Co $(OH)_2$ NSs; **(b)** $Co_{0.2}Ni_{0.8}(OH)_2$ NSs; and **(c)** $Co(OH)_2$ NSs. XPS **(d)** Co 2p and **(e)** Ni 2p spectra of NSs**.**

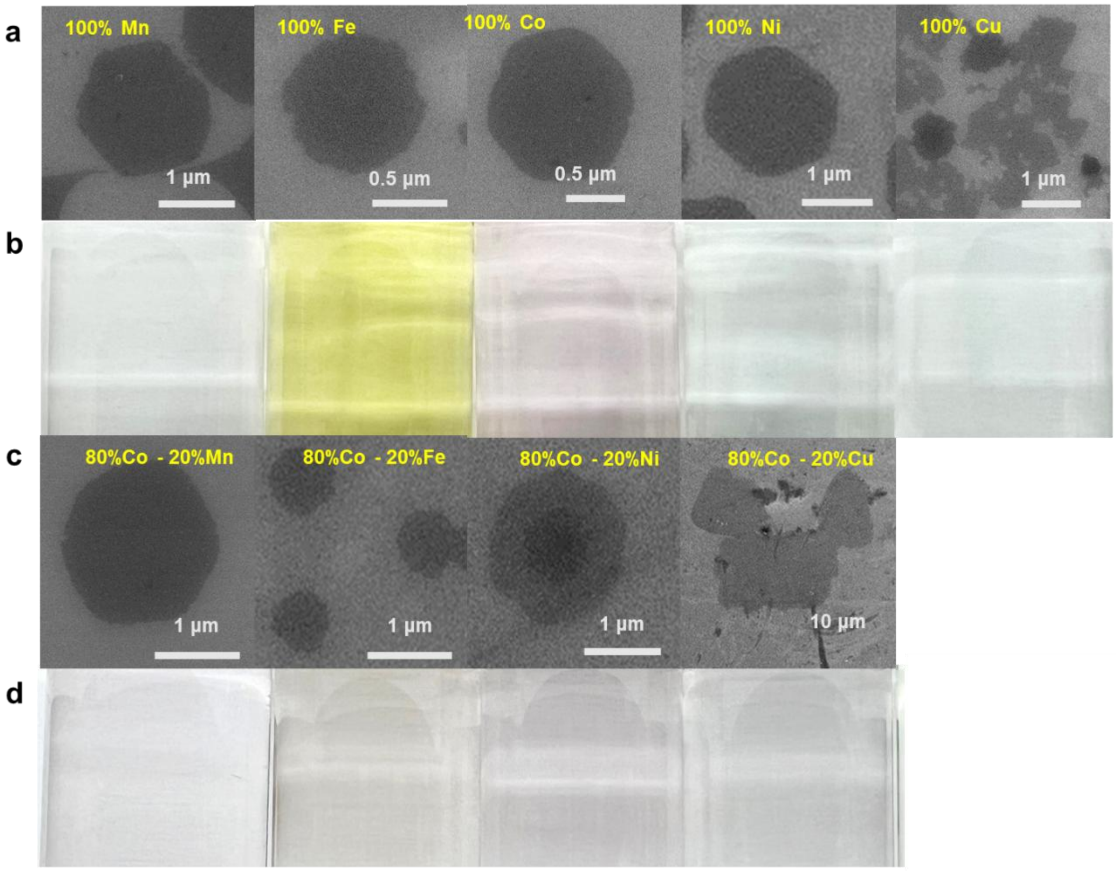

Figure S11. Images of different elements NSs. (a) SEM images of the single-element NSs. **(b)** Optical image of the single-element precursor solutions. **(c)** SEM images of the multi-element NSs. **(d)** Optical image of the multi-element precursor solutions.

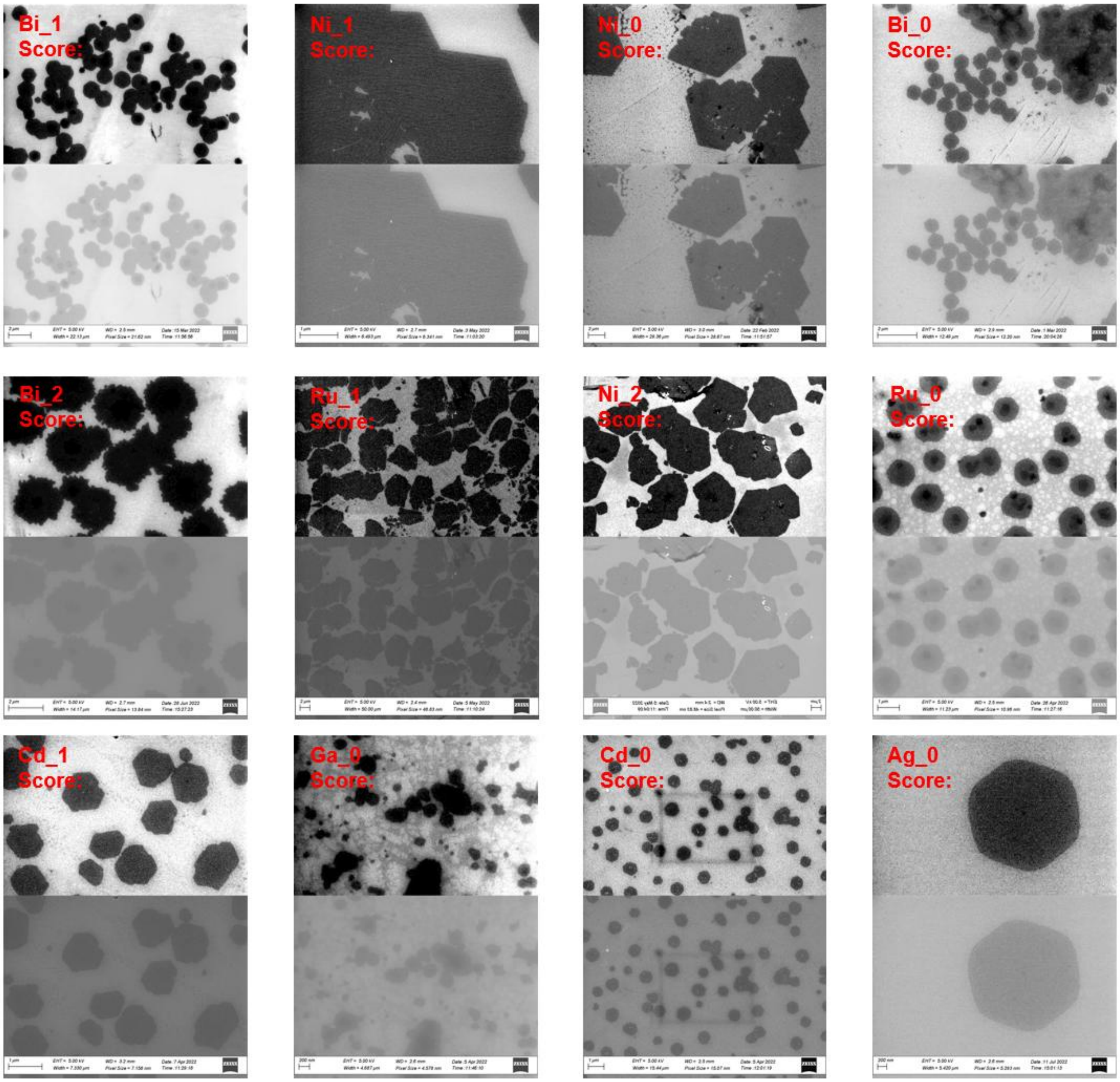

Figure S12. SEM images used to train the LLM

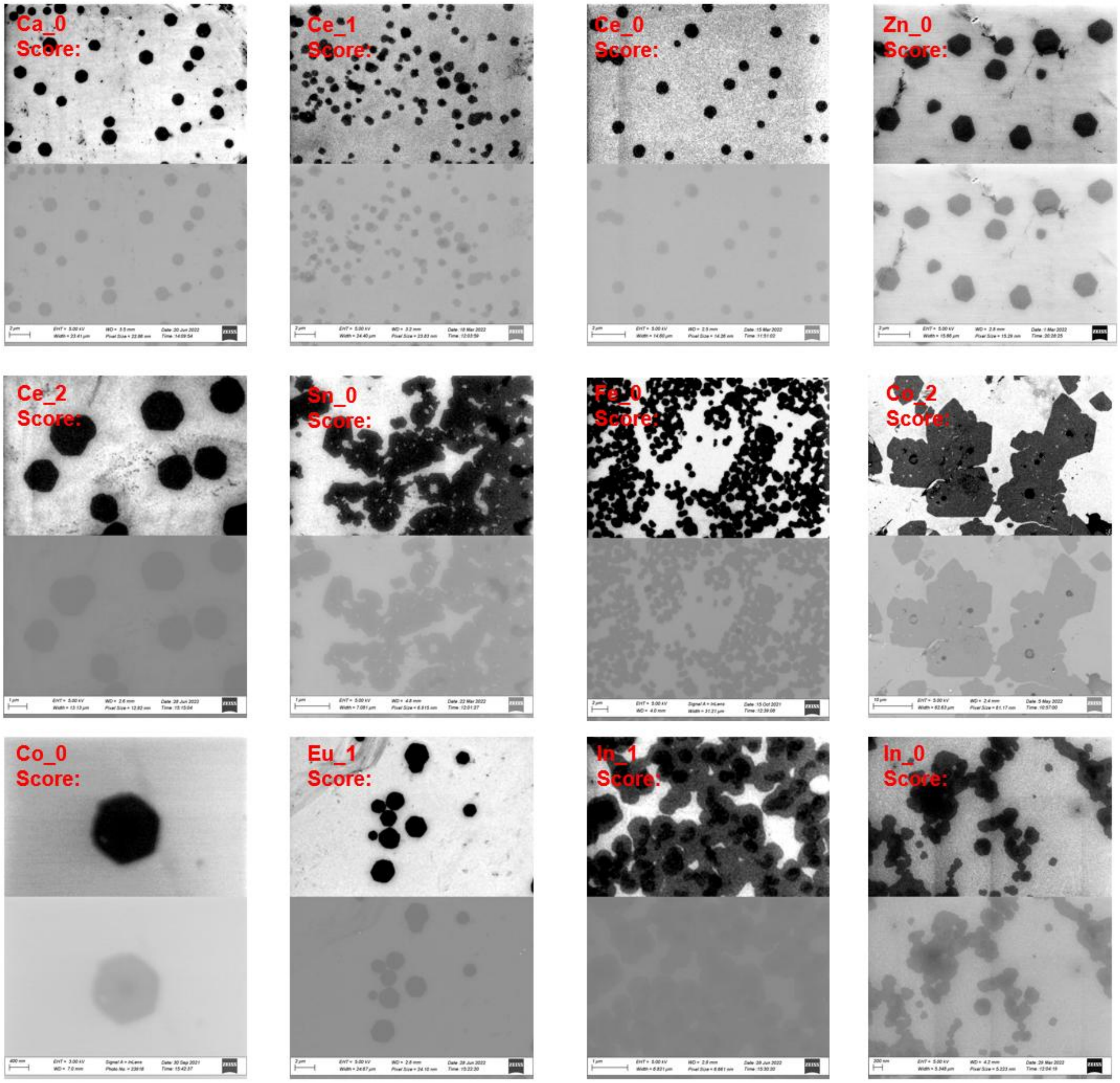

Figure S13. SEM images used to train the LLM

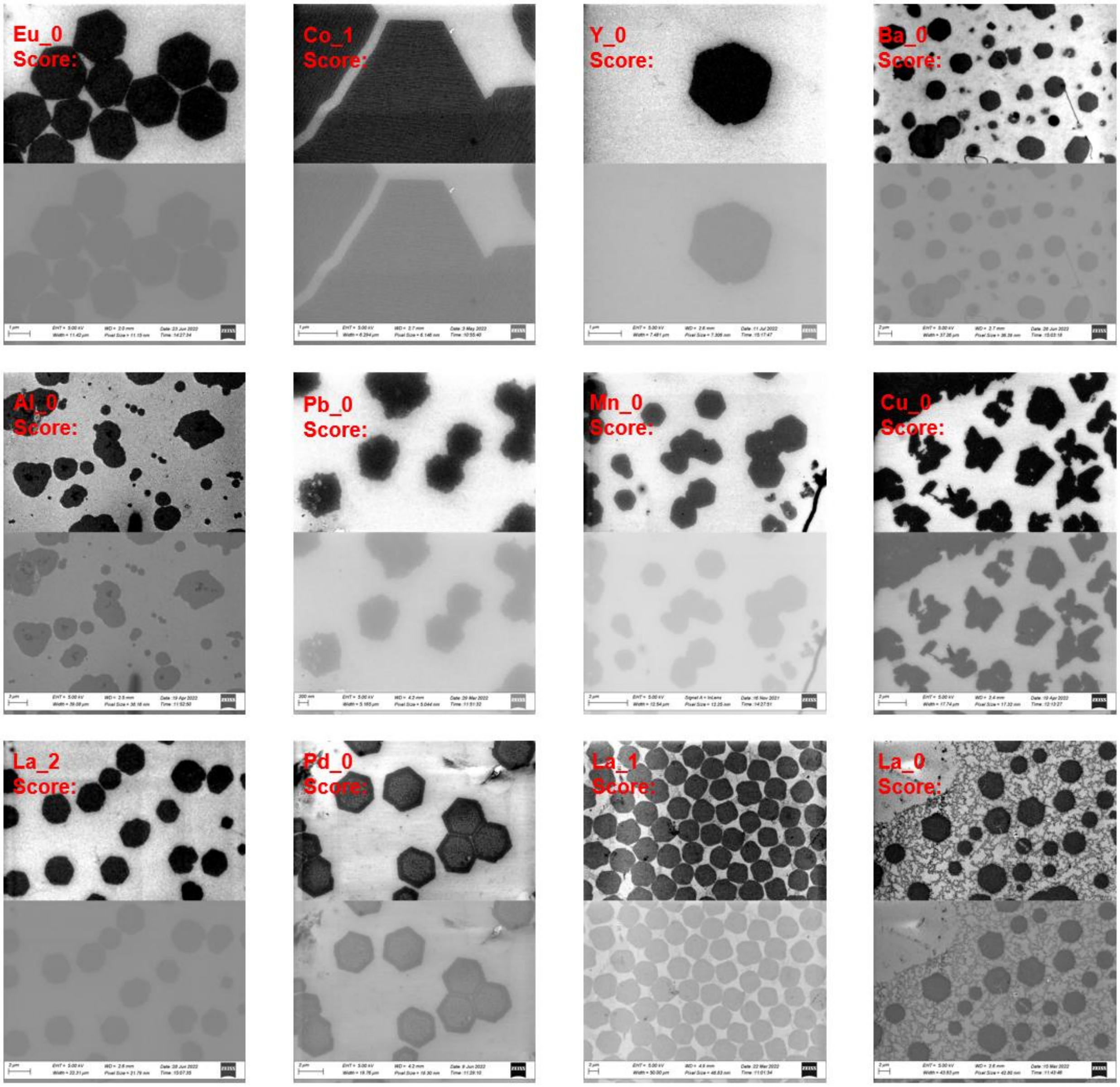

Figure S14. SEM images used to train the LLM

Table 1. ILE Synthetic conditions of hexagonal NSs for different elements.

Atomic #	Element	Metal ion	Tartrate (mM)	ODAM (nmol cm^{-2})	Time (hour)
13	Al	2mM AlCl3	6	1.8	2
20	Ca	5mM CaCl2	4	2.7	4
23	V	0.01mM NH4VO3	2	2.7	2
25	Mn	2.5mM MnCl2	8	1.8	4
26	Fe	2.5mM FeCl2	6	1.8	4
27	Co	2.5mM CoCl2	4	1.8	4
28	Ni	2.5mM NiCl2	2	1.8	4
29	Cu	2.5mM CuCl2	1	3.6	4
30	Zn	1mM ZnCl2	2	2.2	3
31	Ga	1 mM Ga(NO3)3	2	2.2	3
39	Y	0.02 mM Y(NO3)3	2	2.7	2
42	Mo	0.01mM (NH4)2MoO4	2	3.6	2
44	Ru	0.02 mM RuCl3	2	2.7	3
46	Pd	0.02mM PdCl2	2	2.7	3
47	Ag	0.02mM AgNO3	2	2.7	2
48	Cd	2 mM Cd(NO3)2	1	2.7	2
49	In	0.02 mM In(NO3)3	2	3.6	4
50	Sn	0.1mM SnCl2	2	2.7	2
56	Ba	0.02 mM BaCl2	2	3.6	4
57	La	0.02 mM La(NO3)3	2	3.6	2
58	Ce	0.02 mM Ce(NO3)3	2	3.6	2
63	Eu	0.02 mM Eu(NO3)3	2	3.6	2
64	Gd	0.02 mM Gd(NO3)3	2	3.6	2
79	Au	0.01mM HAuCl4	2	2.7	2
82	Pb	0.02 mM PbCl2	2	2.7	2
83	Bi	0.2 mM Bi(NO3)3	1	2.2	6

Table 2. ILE Synthetic conditions of hexagonal NSs for LLM training. (* Solution regulation of readily hydrolysable metal salts.)

Element	Serial number	Metal ion	Tartrate (mM)	ODAM (nmol cm^{-2})	Time (hour)	Solution*
Bi	1	0.2mM Bi(NO3)3*5H2O	1	2.2	6	
Ni	1	10 mM NiCl2	8	2.7	3	
Ni	0	5mM NiCl2	2	2.7	4	10uL 100mM HCl
Bi	0	0.25mM Bi(NO3)3*5H2O	2	2.2	8	
Bi	2	0.02 mM Bi(NO3)3	6	3.6	4	
Ru	1	0.01 mM RuCl3	2	2.7	3	1.6uL 37%HCHO (2mM)
Ni	2	10 mM NiCl2	8	2.7	3	
Ru	0	0.02 mM RuCl3	2	3.6	1	
Cd	1	2 mM Cd(NO3)2	1	2.7	2	
Ga	0	1 mM Ga(NO3)3	2	2.2	3	
Cd	0	1 mM Cd(NO3)2	2	2.2	3	
Ag	0	0.01mM AgNO3	2	2.7	2	
Ca	0	5 mM CaCl2	8	2.7	2	
Ce	1	0.1mM Ce(NO3)3*6H2O	1	2.2	18	
Ce	0	0.2mM Ce(NO3)3*6H2O	1	2.2	6	
Zn	0	1mM ZnCl2	1	2.2	4	
Ce	2	0.02 mM Ce(NO3)3	2	3.6	4	
Sn	0	0.1mM SnCl2	1	2.7	3	
Fe	0	5mM FeCl2	8	3.6	1	
Co	2	10mM CoCl2*6H2O	16	2.7	3	
Co	0	5mM CoCl2*6H2O	4	3.6	2	
Eu	1	0.02 mM Eu(NO3)3	2	3.6	4	
In	1	0.02 mM In(NO3)3	2	3.6	4	
In	0	0.2 mM In(NO3)3	2	2.7	17.5	
Eu	0	0.01 mM Eu(NO3)3	2	2.7	4	
Co	1	10mM CoCl2*6H2O	16	2.7	3	
Y	0	0.02mM Y(NO3)3	2	2.7	2	
Ba	0	0.02 mM BaCl2	2	3.6	4	
Al	0	2 mM AlCl3*6H2O	6	1.8	2	
Pb	0	0.2 mM PbCl2	2	2.7	17.5	
Mn	0	5mM MnCl2*4H2O	16	2.2	4	100uL 100mM NaOH
Cu	0	5mM CuCl2*2H2O	2	1.8	18	
La	2	0.02 mM La(NO3)3	2	3.6	4	
Pd	0	0.01mM PdCl2	2	2.7	4	
La	1	0.1mM La(NO3)3*6H2O	0.5	2.7	3	
La	0	0.2mM La(NO3)3*6H2O	1	2.2	6	

Supplemental Materials on LLMs

Data collection

In this section, we focus on evaluating the quality of grown structures based on their SEM images. While this task may appear straightforward for an expert, the results are often affected by several challenges. The challenges here are representative of those that exist in many tasks associated with hand labeling of complex data in materials science and other fields (e.g., similar issues exist in labeling radiation induced defects in electron microscopy images of irradiated materials[1,2]) Here we list some of the challenges that are faced in general by such data labeling tasks.

1. Efficiency: It takes time to train experts. Such training is typically needed even when following clear guidelines and often requires careful benchmarking efforts to align approaches across multiple labelers. It also takes significant time to do labeling. Scoring large numbers of structures is slow and tedious, leading to fatigue and degraded scoring quality over time.

2. Accuracy: Experts make mistakes even when being careful, and this can get worse when more labeling is required, and people get tired.

3. Consistency: Different experts may score the same structures differently, even when guided by well-defined criteria and careful benchmarking against each other. Even the same expert, when shown the same images at different times, may provide inconsistent scores.

4. Scalability: Many experimental methods are achieving extraordinary scales of thousands of samples and images or more, generally through rapid and automated synthesis and/or characterization methods. Human labeling is already or will soon be unable to scale to manage these large datasets, particularly if the data are produced in real-time and one is targeting rapid or even real-time analysis.

These issues are amplified when a non-expert, relying only on a set of predefined criteria, performs the analysis. This highlights the need for a universal scoring system capable of evaluating structures consistently, objectively, and at high throughput.

Machine learning models for image analysis present promising tools to support researchers in tasks such as determining sample quality [3]. Models like YOLO [4,5] or convolutional neural networks (CNN), which can be trained to identify objects in images, have already been applied in materials research for tasks like defect detection [6,7]. Similarly, Meta's Segment Anything Model (SAM) [8] can identify and segment objects in images without additional training. However, while

these models are useful for object identification or classification, they are not designed to score objects based on subjective criteria. A reliable scoring approach would require extensive training on hundreds or thousands of manually labeled images.

The emergence of multimodal large language models (LLMs) that can analyze various forms of information, including images, opens a new pathway[9]. These advanced models, such as GPT-4, are pre-trained on vast data sets comprising both textual and visual data. This extensive training enables them to understand and generate human-like responses across different modalities, effectively bridging the gap between language and vision.

One of the key advantages of multimodal LLMs is their ability to interpret images in the context of detailed textual descriptions. Instead of relying solely on predefined labels or extensive retraining on specific datasets, these models can analyze an image based on criteria provided in natural language. For instance, researchers can input a comprehensive description of what constitutes a high-quality sample, including subjective aspects that are difficult to quantify objectively. The LLM can then evaluate new images against these criteria, generating scores or assessments that reflect nuanced judgments typically requiring human expertise.

This capability is particularly valuable for tasks where subjective evaluation is essential but hard to standardize. If the LLM can be used high-throughput analysis becomes feasible since the model can process and evaluate large volumes of images efficiently, accelerating research workflows.

In this work, we explore whether this approach can yield a universal, unbiased, and high throughput scoring method. To evaluate this, we selected 36 SEM images of nanosheets grown under various conditions, representing a wide range of structural qualities. These images vary in terms of visual quality (e.g., sharpness, contrast), which are not directly related to the actual quality of the grown structures. To ensure fair assessment, we applied an image post-processing step to normalize contrast and sharpness using well-established image processing techniques (detailed in the Methods section). This step also removed any stamps or labels on the images to provide a clean, unobstructed view of the structures. The final set of 36 normalized images serves as the main dataset for our analysis.

The key metric used to assess the consistency of scoring is the intraclass correlation coefficient (ICC), which measures the correlation between different sets of scores. This allows us to compare consistency across any number of scorers. For a full description of ICC see the Methods section.

To establish a baseline, we conducted scoring with human experts. All participants have extensive experience in the growth and characterization of structures similar to those in the study and were trained within the same lab. Initially, two experts scored the images based on general criteria discussed previously but not explicitly defined. The ICC(3) of their scores was 76%, which we considered surprisingly low, prompting a more structured assessment. To address this, we defined a set of specific criteria for scoring:

1. Edge straightness - edges should be as straight as possible, no wavy or fluffy edges, no attachments.
2. Angles between each edge should be 120 or 60 degrees - structures are supposed to be hexagonal.
3. Corners (corresponding to the angles) - corners should be well defined (not rounded).
4. Symmetry - structures should be highly symmetric and have symmetric features.
5. Consistency - structures should be consistent in shape, size, and surface among other nanosheets.
6. Uniformity - structures should have uniform surfaces (no pits or islands).
7. Isolation - best structures are isolated hexagonal flakes.
8. Edge length difference - edges should be of equal length.

These criteria were then provided to six experts (including the initial two), together with 3 example images of bad, good, and medium quality structures together with their corresponding scores, to establish a baseline for consistency. To test whether a multimodal LLM can serve as an automated scorer, we provided the model with the same set of information as the human scorers. This means we utilized a few-shot approach, where we first showed GPT-4.1 the three example images and a prompt outlining the scoring criteria and then asked to score the whole set of 36 images.

All data, including expert scores, transcripts of GPT-4.1 conversations (with images and full responses, seed, and system fingerprints to allow reproducibility), as well as codes for image processing and result analysis are included with this supplementary information. The latest version

(at the time of submission) of the OpenAI API (1.84.0) and the latest snapshot GPT-4.1 model (gpt-4.1-2025-04-14) have been used.

Methods

The Intraclass Correlation Coefficient (ICC (3)) is used in a two-way mixed-effects model where the images are considered random effects, and the raters are considered fixed effects. (**Figure 7**) It measures the consistency of ratings provided by different raters when systematic differences between raters are acceptable (i.e., raters may have different scoring scales but rank images similarly).

A higher ICC (3) value (closer to 1) indicates greater consistency among the raters' scores across images, reflecting that images maintain their relative rankings regardless of which rater evaluates them.

According to a reported intraclass correlation coefficient giudance[10], the correlation values can be categorized as poor for ICC values less than 0.5, moderate for values between 0.5 and 0.75, good for values between 0.75 and 0.9, and excellent for values greater than 0.90.

$$\text{ICC (3)} = \frac{\text{MS}_{\text{images}} - \text{Ms}_{\text{error}}}{\text{MS}_{\text{images}} + (k-1) \times \text{MS}_{\text{error}}} \quad (1)$$

Where:

$$\text{MS}_{\text{images}} = \frac{\text{SS}_{\text{images}}}{\text{df}_{\text{images}}} = \frac{\sum_{i=1}^{n} k(\bar{Y}_{i\cdot} - \bar{Y}_{\cdot\cdot})^2}{n-1}$$

$$\text{MS}_{\text{error}} = \frac{\text{SS}_{\text{error}}}{\text{df}_{\text{error}}} = \frac{\sum_{i=1}^{n}\sum_{j=1}^{k}(Y_{ij} - \bar{Y}_{i\cdot})^2}{n(k-1)}$$

And:

$$\bar{Y}_{i\cdot} = \frac{1}{k}\sum_{j=1}^{k} Y_{ij} \quad \text{(mean score for image } i\text{)}$$

$$\bar{Y}_{\cdot\cdot} = \frac{1}{nk}\sum_{i=1}^{n}\sum_{j=1}^{k} Y_{ij} \quad \text{(grand mean)}$$

n = number of images k = number of raters

SEM Image Processing

SEM images taken directly off the microscope are of varying quality, in particular the contrast and brightness vary significantly, which makes SEM images often suffer from low contrast, varying brightness, and high levels of noise due to the nature of electron imaging and the thin specimens required for SEM analysis. Important microstructural details may occupy only a narrow range of grayscale intensities and can be obscured by noise or background variations.

To mitigate these issues and make the analysis easier for both the expert scorers and the LLM, we developed an image processing algorithm to normalize the images by enhancing the contrast and brightness by focusing on the most significant intensity ranges - the substrate and the grown structures. First, a denoising filter, specifically the fast Non-local Means Denoising algorithm from OpenCV, is applied to reduce noise while preserving important details. The algorithm then constructs a histogram of the grayscale intensities with a defined bin width to capture the distribution of pixel values. To concentrate on the dominant intensity ranges, histogram bins with counts below a certain threshold are disregarded. The lower and upper intensity thresholds are determined based on the remaining significant bins. Pixels with intensities below the lower threshold are set to black, and those above the upper threshold are set to white, effectively increasing the contrast by stretching the middle intensity values across the full grayscale spectrum. The intensities within the significant range are linearly scaled, enhancing features that are most prominent in the image. The full code for image processing is available in Supplementary Information.

References:

1 R. Jacobs, M. Shen, Y. Liu, W. Hao, X. Li, R. He, J. R. Greaves, D. Wang, Z. Xie, Z. Huang, C. Wang, K. G. Field, and D. Morgan, Performance and limitations of deep learning semantic segmentation of multiple defects in transmission electron micrographs, Cell Reports Phys- ical Science 3, 100876 (2022).

2 R. Jacobs, P. Patki, M. J. Lynch, S. Chen, D. Morgan, and K. G. Field, Materials swelling revealed through automated semantic segmentation of cavities in electron microscopy images, Scientific Reports 13, 5178 (2023).

3 M. Ragone, R. Shahabazian-Yassar, F. Mashayek, and V. Yurkiv, Deep learning modeling in microscopy imaging: A review of materials science applications, Progress in Materials Science 138, 101165 (2023).

4 J. Redmon, S. Divvala, R. Girshick, and A. Farhadi, You only look once: Unified, real-time object detection (2016), arXiv:1506.02640 [cs.CV].

5 J. Terven, D.-M. C´ordova-Esparza, and J.-A. RomeroGonza´lez, A comprehensive review of yolo architectures in computer vision: From yolov1 to yolov8 and yolo-nas, Machine Learning and Knowledge Extraction 5, 1680(2023).

6 M. Shen, G. Li, D. Wu, Y. Yaguchi, J. C. Haley, K. G. Field, and D. Morgan, A deep learning based automatic defect analysis framework for in-situ tem ion irradiations, Computational Materials Science 197, 110560 (2021).

7 G. Roberts, S. Y. Haile, R. Sainju, D. J. Edwards, B. Hutchinson, and Y. Zhu, Deep learning for semantic segmentation of defects in advanced stem images of steels, Scientific Reports 9, 12744 (2019).

8 A. Kirillov, E. Mintun, N. Ravi, H. Mao, C. Rolland,L. Gustafson, T. Xiao, S. Whitehead, A. C. Berg, W.-Y.Lo, P. Doll´ar, and R. Girshick, Segment anything (2023), arXiv:2304.02643 [cs.CV].

9 OpenAI, Gpt-4 technical report (2024), arXiv:2303.08774 [cs.CL].

10 T. K. Koo and M. Y. Li, A guideline of selecting and reporting intraclass correlation coefficients for reliability research, Journal of Chiropractic Medicine 15, 155 (2016).
